# The genetic relationship between female reproductive traits and six psychiatric disorders

**DOI:** 10.1038/s41598-019-48403-x

**Published:** 2019-08-19

**Authors:** Guiyan Ni, Azmeraw T. Amare, Xuan Zhou, Natalie Mills, Jacob Gratten, S. Hong Lee

**Affiliations:** 1 Institute for Molecular Bioscience, University of Queensland, Brisbane, Queensland 4072 Australia; 2 Australian Centre for Precision Health, University of South Australia Cancer Research Institute, University of South Australia, Adelaide, SA 5000 Australia; 3 School of Environmental and Rural Science, University of New England, Armidale, NSW 2351 Australia; 4 South Australian Academic Health Science and Translation centre, South Australian Health and Medical Research Institute (SAHMRI), Adelaide, South Australia Australia; 5 Discipline of Psychiatry, School of Medicine, University of Adelaide, Adelaide, SA Australia; 6 Mater Research Institute, University of Queensland, Brisbane, Queensland 4072 Australia

**Keywords:** Genomics, Genetic association study

## Abstract

Female reproductive behaviours have important implications for evolutionary fitness and health of offspring. Here we used the second release of UK Biobank data (N = 220,685) to evaluate the association between five female reproductive traits and polygenic risk scores (PRS) projected from genome-wide association study summary statistics of six psychiatric disorders (N = 429,178). We found that the PRS of attention-deficit/hyperactivity disorder (ADHD) were strongly associated with age at first birth (AFB) (genetic correlation of −0.68 ± 0.03), age at first sexual intercourse (AFS) (−0.56 ± 0.03), number of live births (NLB) (0.36 ± 0.04) and age at menopause (−0.27 ± 0.04). There were also robustly significant associations between the PRS of eating disorder (ED) and AFB (0.35 ± 0.06), ED and AFS (0.19 ± 0.06), major depressive disorder (MDD) and AFB (−0.27 ± 0.07), MDD and AFS (−0.27 ± 0.03) and schizophrenia and AFS (−0.10 ± 0.03). These associations were mostly explained by pleiotropic effects and there was little evidence of causal relationships. Our findings can potentially help improve reproductive health in women, hence better child outcomes. Our findings also lend partial support to the evolutionary hypothesis that causal mutations underlying psychiatric disorders have positive effects on reproductive success.

## Introduction

Female reproductive behaviours, including age at first birth (AFB), age at first sexual intercourse (AFS), age at menarche (AMC), age at menopause (AMP) and number of live births (NLB) have important implications in reproductive health and evolutionary fitness^1,2^. Some of these traits have been shown to associate with the physical and mental health of offspring^3^, and there has been growing evidence that maternal AFB is associated with increased risk of psychiatric disorder^4,5^ and behavioural problems in their children^6,7^. For instance, both early and late maternal AFB are associated with an increased risk to schizophrenia (SCZ)^4^, bipolar disorder (BIP)^8^, attention-deficit/hyperactivity disorder (ADHD)^7^, autism spectrum disorder (ASD)^9^ and depression^10^ in offspring. Age at menopause and menarche also tend to correlate with the risk of adverse mental health outcomes in offspring^11–14^.

The relationship between reproductive behavior and susceptibility to psychiatric disorders is likely complex and bidirectional. Individuals with psychiatric illnesses and their relatives may be more prone to risk taking and impulsive behaviors, which cause early pregnancy and childbirth in women, or they may exhibit poor social interactions that result in delays in key reproductive transitions such as marriage, pregnancy and childbirth^15–20^.

In addition to the epidemiological evidence, the phenotypical relationship between female reproductive traits and psychiatric disorder risk in offspring may have a genetic basis^21–26^. Genome wide association studies (GWAS) that examined reproductive behaviors and psychiatry disorders separately have identified several genome-wide significant genetic loci influencing AFB^21^, NLB^21^ and AFS^3^, and ones affecting susceptibility to ADHD^27^, ASD^23^, eating disorders (ED)^28^, BIP^29^, major depressive disorder (MDD)^30^, and SCZ^31^. When reproductive traits and psychiatric disorders are examined together, a complex genetic relationship between the two clusters of traits begins to emerge^32^. For instance, recent studies^24,26^ showed a U-shaped relationship between AFB and the polygenic risk score of SCZ^24^, which echoes with the phenotypic association between maternal age and SCZ risk in offspring found in epidemiological studies (e.g. McGrath *et al*.^4^, El-Saadi *et al*.^33^, Byrne *et al*.^34^). However, for most other psychiatric disorders, the latent genetic architecture shared with reproductive traits remains unknown.

In this study, we investigate the genetic association of five specific reproductive traits in women (AFB, AFS, AMC, AMP and NLB) with six common psychiatric disorders (ADHD, ASD, ED, BIP, MDD, and SCZ) using polygenic score and LDSC approach. Causal relationship between the risk of psychiatric disorders and female reproductive traits is further tested using Mendelian randomization. This will shed light on the complex bio-psychosocial risk factors associated with shared genetic effects between female reproductive and psychiatric traits. Furthermore, this study provides insight into whether complex phenotypes of reproductive behaviour can be caused by mental health or rather by pleiotropic genes associated with both traits.

## Materials and Methods

UK Biobank’s scientific protocol and operational procedures were reviewed and approved by the North West Multi-centre Research Ethics Committee (MREC), National Information Governance Board for Health & Social Care (NIGB), and Community Health Index Advisory Group (CHIAG). UK Biobank also obtained informed consent from the study participants. Research Ethics approval was obtained from University of South Australia Human Research Ethics Committee (HREC). All methods were performed in accordance with the relevant guidelines and regulations.

### Data

#### UK Biobank sample and quality control

We used the second release of the UK Biobank dataset that initially included 264,859 women with imputed genotypes for 35.6 million SNPs. The imputation was based on the Haplotype Reference Consortium reference panel^35^. The quality control (QC) criteria for the imputed genotype data included an imputation reliability (INFO score) >0.6^36–38^, minor allele frequency >0.01, p-value for Hardy-Weinberg equilibrium test >10E-7, and SNP missingness <0.05. Women with non-white British ancestry, individual missingness >0.05, individuals with putative sex chromosome aneuploidy or one individual per pair of relatives was excluded. After the QC above, we compared discordance between the first and second release of QCed UK Biobank data for each SNP and individual that were excluded if the discordance rate was above 0.05. In addition, we excluded genome-wide significant SNPs in a GWAS analysis where individuals in the first and second release UK Biobank data were treated as cases and controls. Subsequently, 220,685 individuals and 7,253,311 SNPs remained, from which 1,133,064 Hapmap3 SNPs were selected for the analyses. Traits of interest were AFB, AFS, AMC, AMP, and NLB. Number of observations for each trait are provided in Table 1.

**Table 1:** Sample breakdown by age at first birth, age first had sexual intercourse, age at menarche, age at menopause and number of live births.

AFB	N	AFS	N	AMC	N	AMP	N	NLB	N
<20	11,414	<16	13,915	<11	8,058	<40	4,055	0	32,805
20 to <25	42,048	16 to <20	86,038	11 to <13	60,029	40 to <46	14,468	1	23,284
25 to <30	46,966	20 to <24	43,054	13 to <15	76,625	46 to <52	42,704	2	80,594
30 to <35	16,880	24 to <28	9,690	15 to <17	25,859	52 to <58	38,011	3	31,182
≥35	4,236	≥28	3,446	≥17	2,285	≥58	3,148	>3	9,879
Total	121,544		156,143		172,856		102,386		177,744

AFB: Age at first birth. AFS: Age at first sexual intercourse. AMC: Age at menarche. AMP: age at menopause. NLB: Number of live births.

#### Psychiatric genomics consortium (PGC) GWAS summary statistics results

The GWAS summary results of six psychiatric disorders were obtained from the PGC (http://www.med.unc.edu/pgc), including ADHD^27^, ASD^23^, ED^28^, BIP^29^, MDD^30^, and SCZ^31^. The number of cases and controls in these studies are provided in Table S1.

### Statistical analyses

#### Estimation of polygenic risk scores (PRS)

For each psychiatric disorder, PRS were computed for the UK Biobank sample as the sum of the risk alleles weighted by the estimated SNP effects from the PGC GWAS that are in public domain (http://www.med.unc.edu/pgc/) and not likely to include UK Biobank sample. To compute PRS for the UK Biobank sample we used approximately 1,133,064 HapMap 3 SNPs from each study. The projection of the SNP effects onto the UK Biobank data was conducted using MTG2^39^.

#### Mean difference of PRS across the five categories

For each reproductive trait, we first divided UK Biobank samples into five groups according to their values on the trait (see Table 1 for sample breakdowns). We then did pairwise comparisons between group means for each trait using two-tailed t-tests.

#### Linear prediction

Using a linear or polynomial regression model, we assessed, for each disorder, if PRS significantly predicted each of the phenotypes of the reproductive traits. In the prediction model, each of the reproductive traits was adjusted for age at interview, year of birth, study center, genotype batch, and the first 15 principal components (PCs) provided by the UK Biobank. For sensitivity analyses, we repeated the prediction using additional variables including educational attainment, income level, and smoking and alcohol consumption status.

#### Genetic correlation

Genetic correlation is a classic population parameter used to infer the geometric mean of trait variance (i.e. the additive genetic covariance between two traits scaled by the square root of the product of the genetic variance for each trait). Pairwise genetic correlations were estimated between the reproductive traits and psychiatric disorders using linkage disequilibrium score regression (LDSC) based on GWAS summary statistics^40,41^. GWAS was carried out and SNP effects were estimated for each of the reproductive traits using PLINK 1.9^42^. In the GWAS, phenotypes were adjusted for age at interview, year of birth, assessment center at which participant consented, genotype batch and the first 15 PCs. We also used additional covariates such as educational attainment, income level, smoking and alcohol consumption status to further adjust the phenotypes in sensitivity analyses.

#### Analysis of overlapping samples between PGC and UK Biobank data

The intercept of LDSC is estimated as ${\varrho }{N}_{s}/\sqrt{{N}_{1}{N}_{2}}$, where ${\varrho }$ is the phenotypic correlation among *N*_*s*_ overlapping individuals in the two studies with sample sizes of *N*_1_ and *N*_2_, respectively^40^. To check if there were any overlapping samples between UK Biobank and PGC, we explicitly estimated the intercept when using LDSC to estimate genetic correlations between the reproductive traits and psychiatric disorders

#### Causal effects of psychiatric disorder PRS on female reproduction traits

We performed two-sample Mendelian randomization (MR) analyses to test whether there were causal relationships between the risk of psychiatric disorders and female reproductive traits. We used psychiatric disorder PRS as exposure data and female reproductive traits as outcomes. Genome-wide significant SNPs (p < 5E-08) from the PGC GWAS summary statistic results were used as instruments in the MR analyses. Associations between the instruments and the outcomes were tested^42^. Prior to the association test, we adjusted the outcomes, the phenotypes of the female reproductive traits, for age at interview, year of birth, assessment centre at which participant consented, genotype batch and the first 15 PCs. We used an inverse-variance weighted (IVW) regression^43^ as the primary analysis to determine a causal relationship and used MR-Egger^44^, weighted median^45^ and weighted mode^46^, which rely on different assumptions regarding the validity of instruments, for sensitivity analyses. Details of those assumptions are described elsewhere^43–46^. MR analyses were performed using a R package, TwoSampleMR^47^, and P-values were obtained using a two-tailed t-test with the respective degree of freedom. The instruments were available for ADHD, BIP and SCZ only, therefore ASD, ED, MDD were not tested for the MR analyses.

## Results

### Basic characteristics

In this study, we analyzed genetic data from 220,685 women who were part of the UK Biobank. The average age of the study participants at recruitment was 57, with a range from 40 to 71. The number of observations for each reproductive trait is provided in Table 1. The total sample of women was divided into five groups according to their AFB, AFS, AMC, AMP or NLB status (Table 1) to detect the mean difference of PRS across the five categories.

### Mean difference of PRS across the five categories

**Figure 1:**
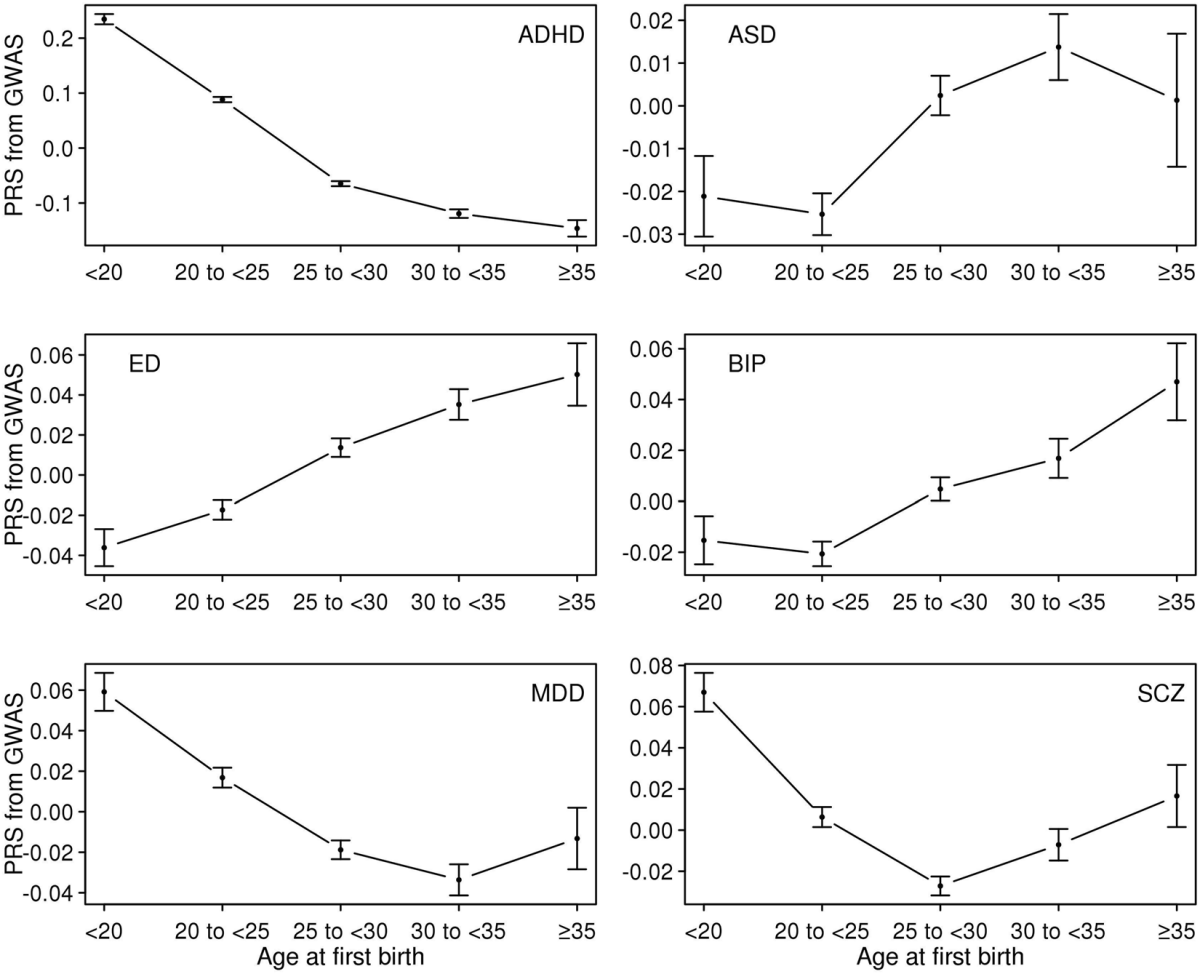
Means and standard errors of PRSs for the six psychiatric disorders, by age at first birth, in the UK Biobank sample.

**Figure 2:**
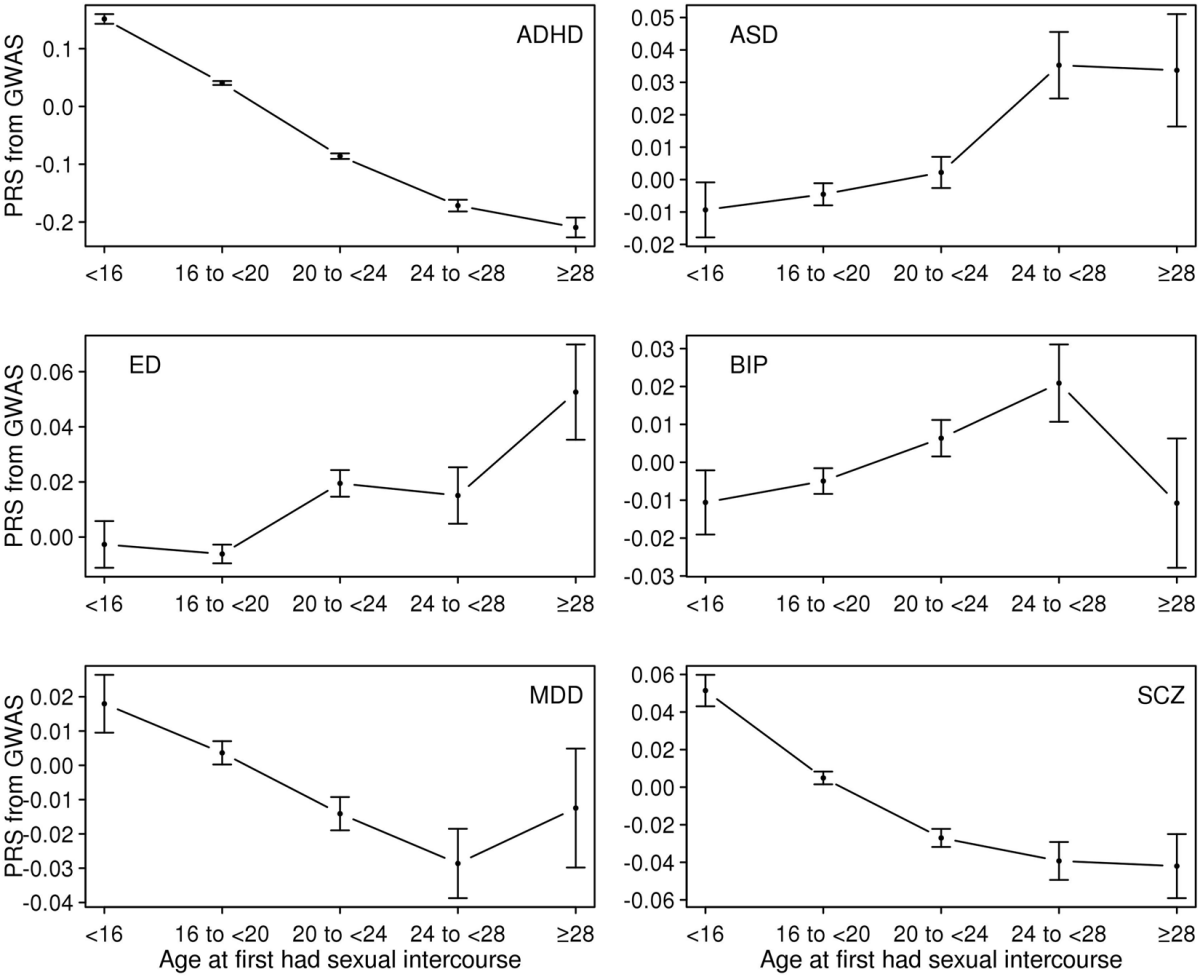
Means and standard errors of PRS for the six psychiatric disorders, by age at first sexual intercourse, in the UK Biobank sample.

**Figure 3:**
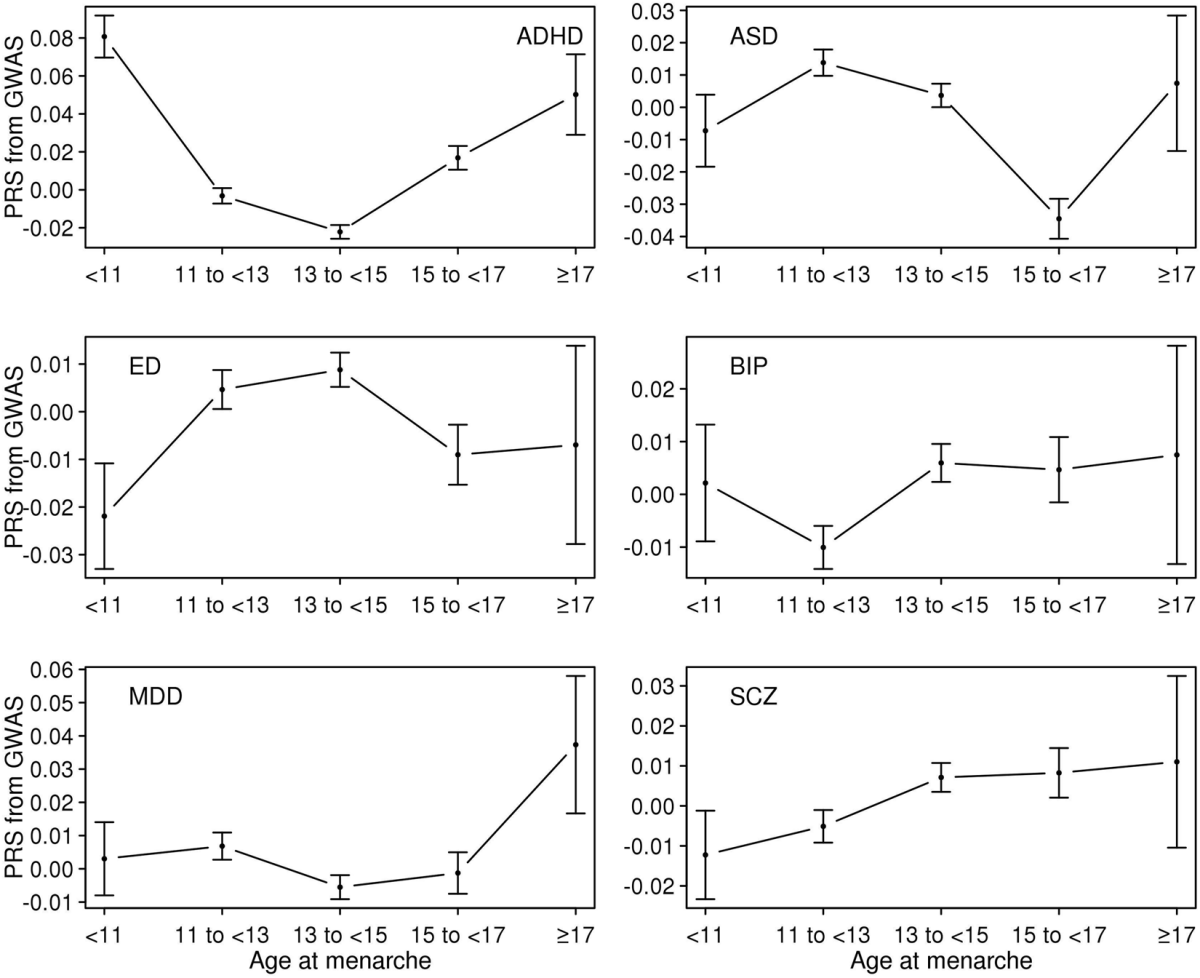
Means and standard errors of PRS for the six psychiatric disorders by age at menarche in the UK Biobank sample.

**Figure 4:**
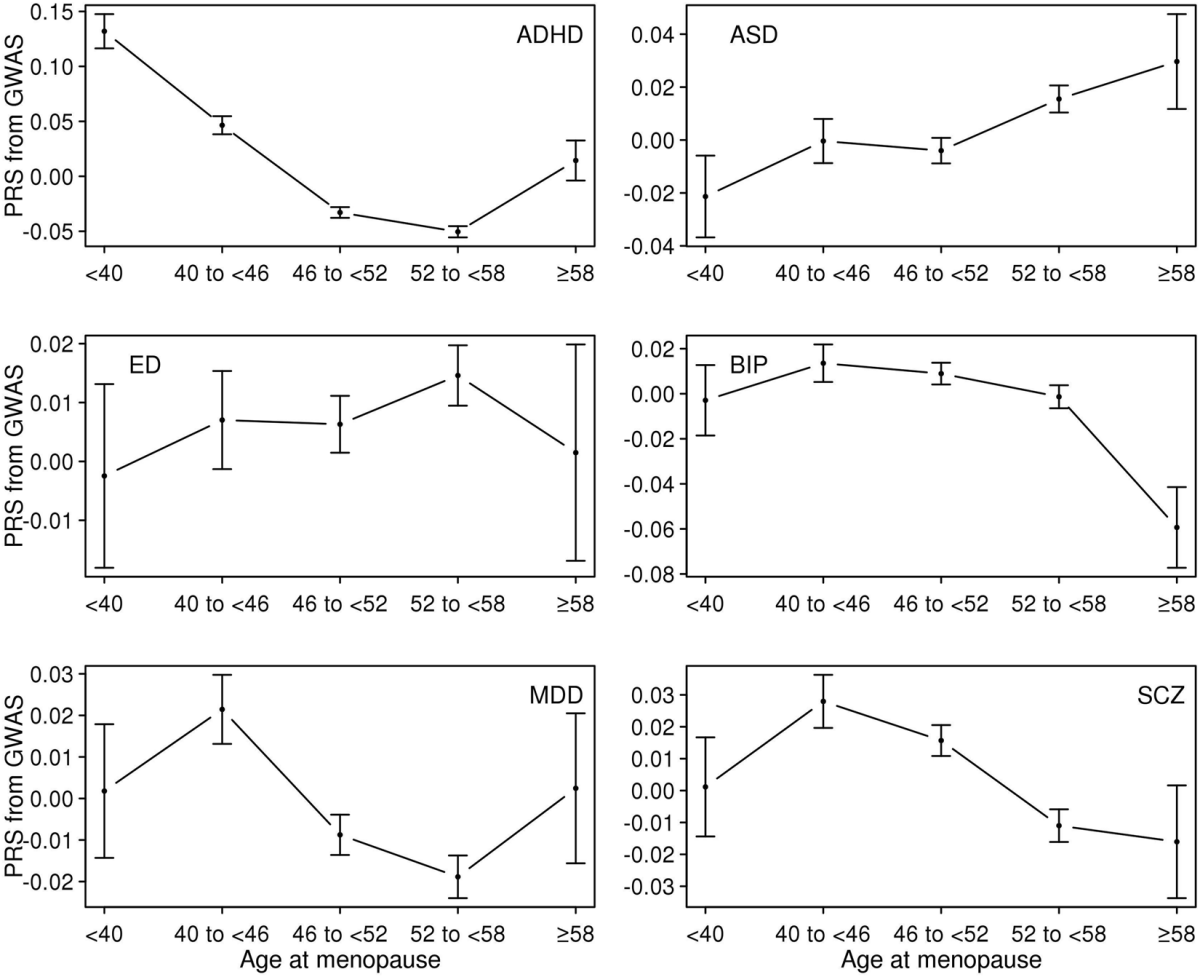
Means and standard errors of PRS for the six psychiatric disorders by age at menopause in the UK Biobank sample.

**Figure 5:**
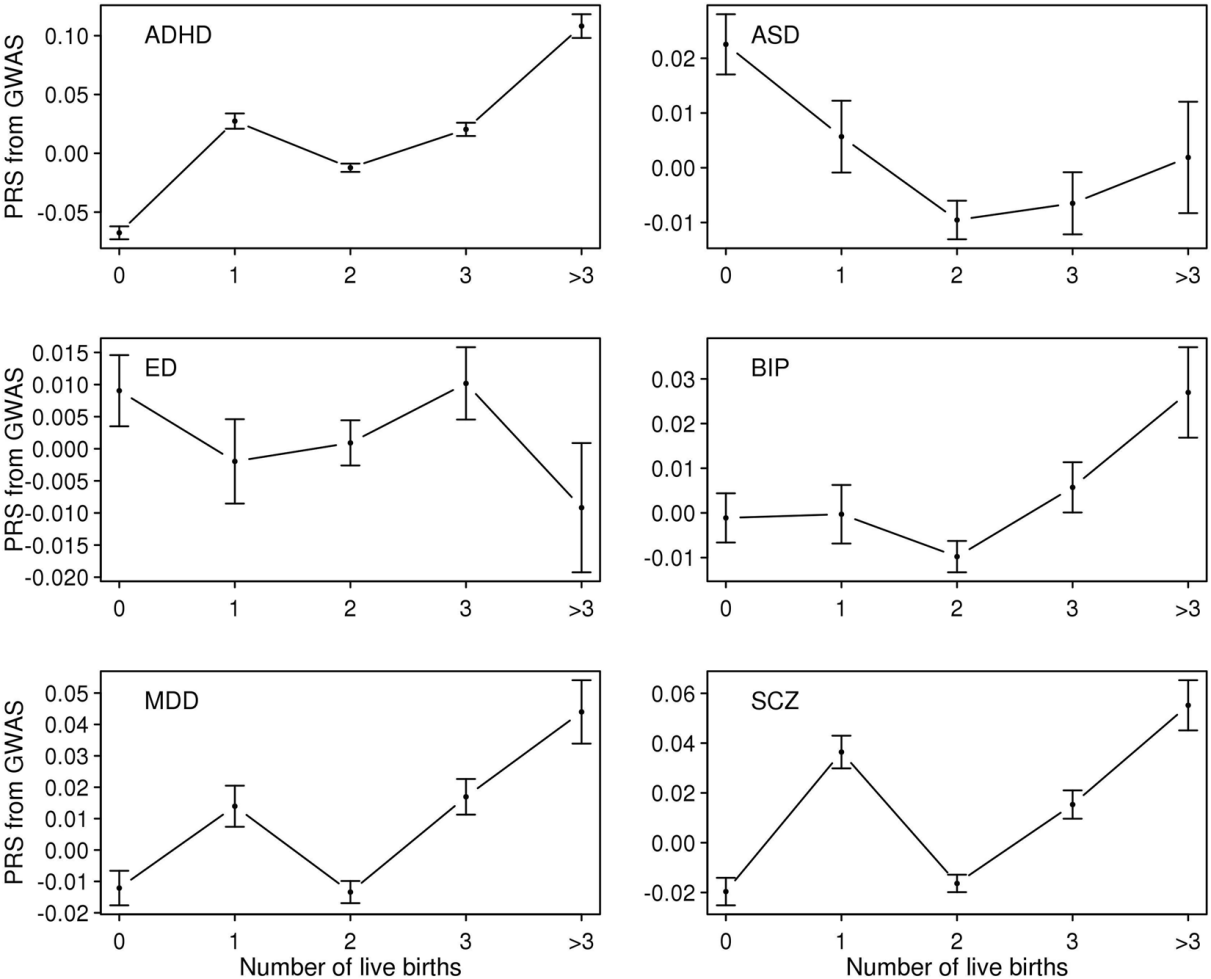
Means and standard errors of PRS for the six psychiatric disorders by number of live births in the UK Biobank sample.

First, we computed PRS of the six psychiatric disorders for the UK Biobank sample and estimated the mean of the PRS for each age category of each reproductive trait (Figs 1–5). Then, the mean difference of the PRS was tested across the five categories for each of the reproductive traits (Tables S2–S6). A statistically significant difference in the PRS was determined after Bonferroni correction for multiple testing (i.e. significance level divided by the number of tests, 0.05/300 = 1.7E-4) and the results that passed the significance threshold were highlighted in bold (see Tables S2–S6).

In Fig. 1, the direction of association between the PRS and AFB was positive (the older the AFB, the higher the risk) for ED, ASD and BIP, but negative (the younger the AFB, the higher the risk) for ADHD, MDD and SCZ although some traits (MDD and SCZ) showed non-linear (U-shaped) associations. A U-shaped relationship was previously shown between AFB and SCZ in an independent study^24^. The mean difference in PRS was statistically significant for most pairwise comparisons of age classes for ADHD, ED and MDD (Table S2). The significance was particularly pronounced for ADHD, with a P-value of 2.0E-184, for example, for the difference between women with AFB < 20 and women with 30 ≤ AFB < 35 (Table S2). For SCZ, most significant signals came from younger AFB groups (Fig. 1 and Table S2).

**Figure 6:**
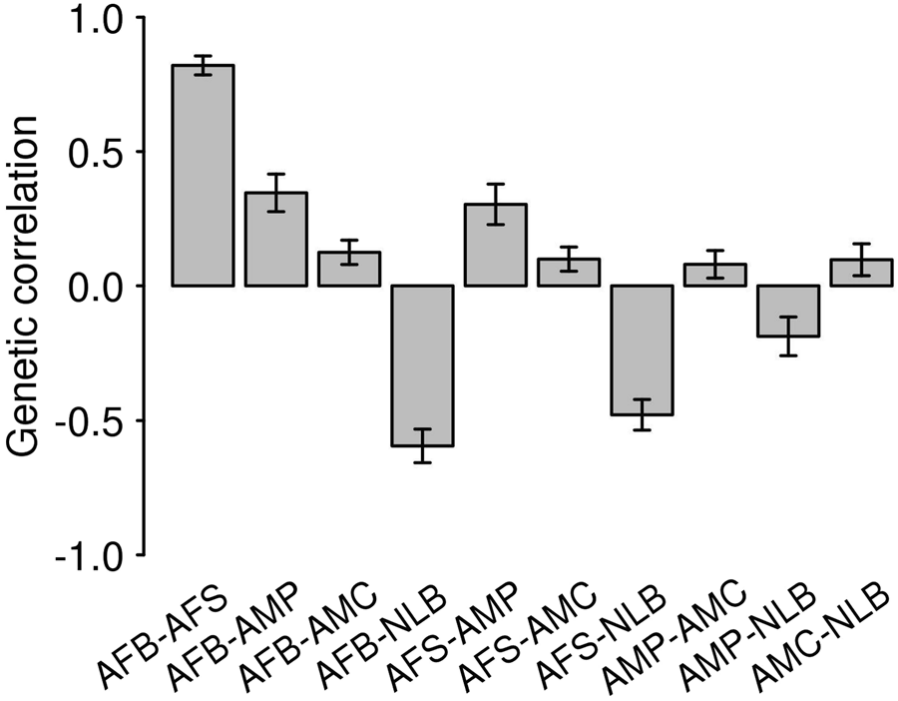
Genetic correlations among the five reproductive traits estimated using the base model. In the base model, the reproductive traits were adjusted for age at interview, year of birth, study center, genotype batch, and the first 15 principal components. Error Bars are 95% confidence intervals. AFB: Age at first birth. AFS: Age at first sexual intercourse. AMC: Age at menarche. AMP: age at menopause. NLB: Number of live births.

The results for AFS were similar to those for AFB, consistent with the strong correlation between these traits (Fig. 6), although some signals were reduced and not significant after the Bonferroni correction (ASD, BIP and MDD) (Fig. 2 and Table S3).

For AMC and AMP, there were relatively few statistically significant differences between age groups for the six psychiatric disorders, with the exception of ADHD (Tables S4 and S5). The relationship between the PRS of ADHD and AMC was non-linear (Fig. 3), with earlier or later AMC tending to have significantly higher ADHD risk than intermediate AMC (Table S4). A similar relationship was observed between the ADHD PRS and AMP in that earlier or later AMP was associated with higher ADHD risk than moderate AMP (Fig. 4 and Table S5).

Since NLB was expected to be negatively correlated with AFB (see Fig. 6), the pattern of the mean PRS of ADHD was inversely correlated between the groups classified according to NLB and AFB (Figs 1 and 5). Table S6 shows that the ADHD PRS was strongly associated with NLB. In addition, there were a number of significant association signals of NLB for MDD and SCZ (Table S6).

### Linear prediction

We used a regression method to assess the significance of genetic association between the reproductive behavior traits and six psychiatric disorders (see Methods).

**Figure 7:**
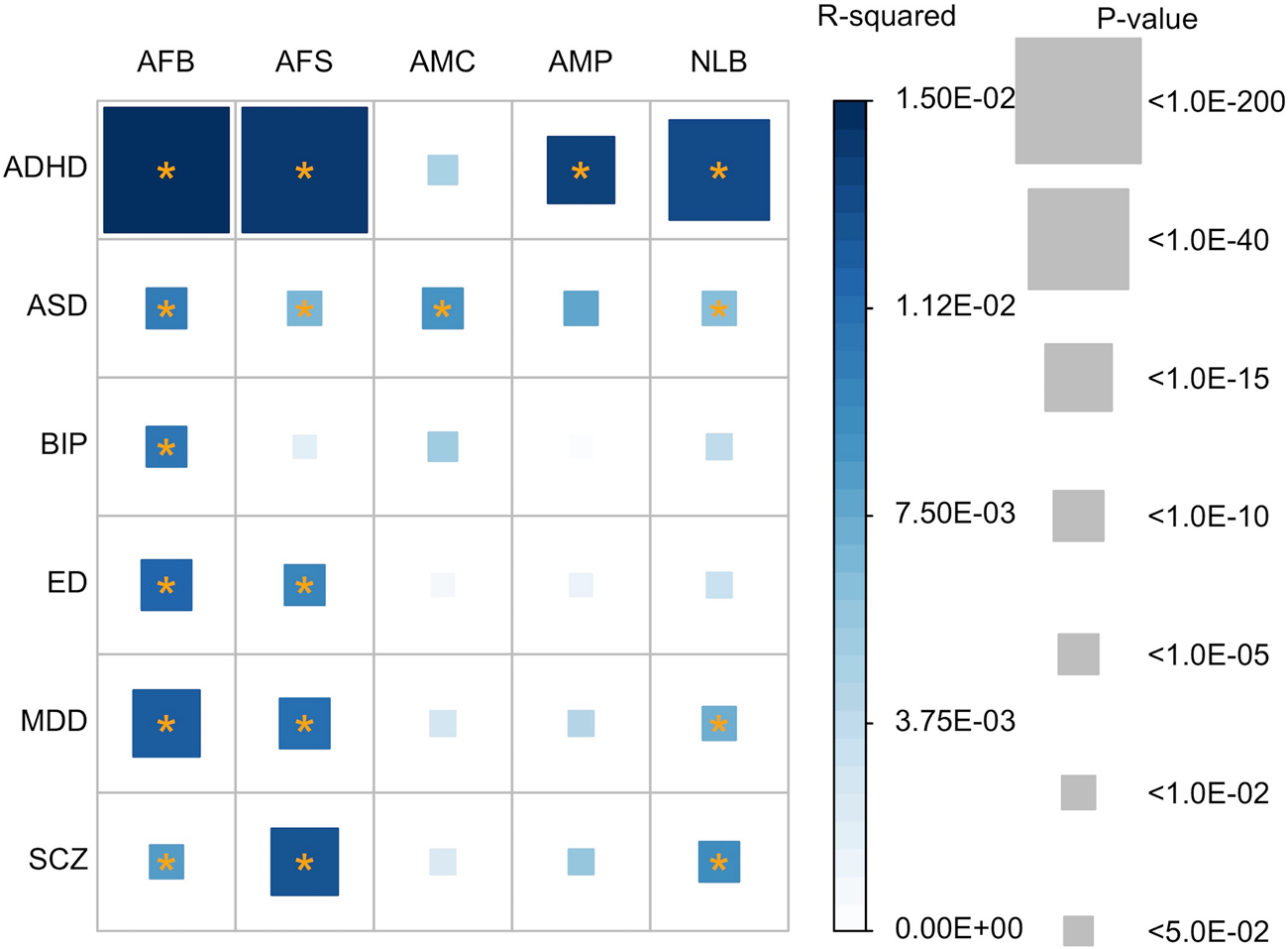
Coefficient of determination (R^2^) and p-values for its significance based on a linear prediction model. Color of each box represents the level of R-squared, and the size of squares represents its significance (p-value). R-squared that are significantly different from zero after Bonferroni correction (0.05/30) are marked with an asterisk. Dependent variables were adjusted for age at interview, year of birth, assessment centre at which the participant consented, genotype batch, and the first 15 principal components. The number of records used for the analyses was 121,544 for AFB, 156,143 for AFS, 102,386 for AMP, and 172,856 for AMC and 177,744 for NLB. AFB: age at first firth. AFS: age at first sexual intercourse. AMP: age at menopause. AMC: age at menarche. NLB: number of live births. ADHD: Attention-Deficit/Hyperactivity Disorder. ASD: Autism spectrum disorder. ED: Eating disorder. BIP: Bipolar disorder. MDD: Major depressive disorder. SCZ: Schizophrenia.

We regressed each of pre-adjusted reproductive traits (AFB, AFS, AMC, AMP and NLB) on the PRS of each of the psychiatric disorders. Using the linear regression prediction modeling, we showed that the PRS of each six psychiatric disorder were significantly associated with at least one of the five reproductive traits, confirming some of the robust associations from the earlier analyses of mean difference of PRS across the five age categories (Tables S2–S6). Of all the five reproductive traits, AFB was the trait best predicted by the PRS of the six psychiatric disorders (Fig. 7, the corresponding R-squared and P-values are in Table S7). Because AFB and AFS are highly correlated traits, the results for AFS were similar to those for AFB except that there was no significant association between PRS of AFS and BIP, which is consistent with the analyses of mean difference of PRS above (Tables S2 and S3). NLB was significantly predicted by the PRS of ADHD, ASD, MDD, and SCZ. In addition, AMC and AMP were only associated with the PRS of ASD and ADHD, respectively. We noted that the majority of significance was explained by linear predictions, but not by quadratic polynomial predictions for all of the associations (Table S8). There were marginal significances for quadratic polynomial associations only for a few pairwise comparisons (AFB vs SCZ and AFS vs ED).

We conducted a sensitivity analysis in which dependent variables were further adjusted for educational attainment, income level, smoking and alcohol consumption status (Fig. S1 and Table S9). Most of the association signals remained significant with some exceptions. For example, the association of AFB, AFS, and NLB with ASD disappeared, as did the association between AFB and BIP. Conversely, ADHD became significantly associated with AMC after correcting for the additional covariates.

When using GWAS p-value thresholds to filter SNPs, the significance of linear prediction decreased for most of the association analyses between the reproductive traits and six psychiatric disorders (Table S10). This indicates that the associations between the reproductive traits and six psychiatric disorders were probably due to many genes, not due to a few major genes.

### Genetic correlations

We used LDSC to estimate genetic correlations between the reproductive behavior traits and six psychiatric disorders (see Methods). We estimated genetic correlations between the five reproductive traits to reveal the shared genetic architecture of the traits (Fig. 6). As expected, the genetic correlation between AFB and AFS was very high (0.821 ± 0.018) and that between AFB and NLB was high (−0.594 ± 0.032). However, the genetic correlation between AMC and AMP was relatively low and they also have moderate or low genetic correlations with other reproductive traits, e.g. 0.346 ± 0.036 between AFB and AMP, 0.303 ± 0.039 between AFS and AMP and ~ 0.1 between AMC and other traits (Fig. 6). These results explain the observations in the analyses of mean difference in PRS (Figs 1–5) and linear predictors (Fig. 7), where the results between AFB and AFS were similar, and those between AFB (AFS) and NLB were reciprocally similar for the association with ADHD PRS. The estimated genetic correlations among those five reproductive traits remained similar after additional adjustment of the dependent variables for educational attainment, income levels, and smoking and alcohol consumption status (Fig. 6 vs. Fig. S2).

**Figure 8:**
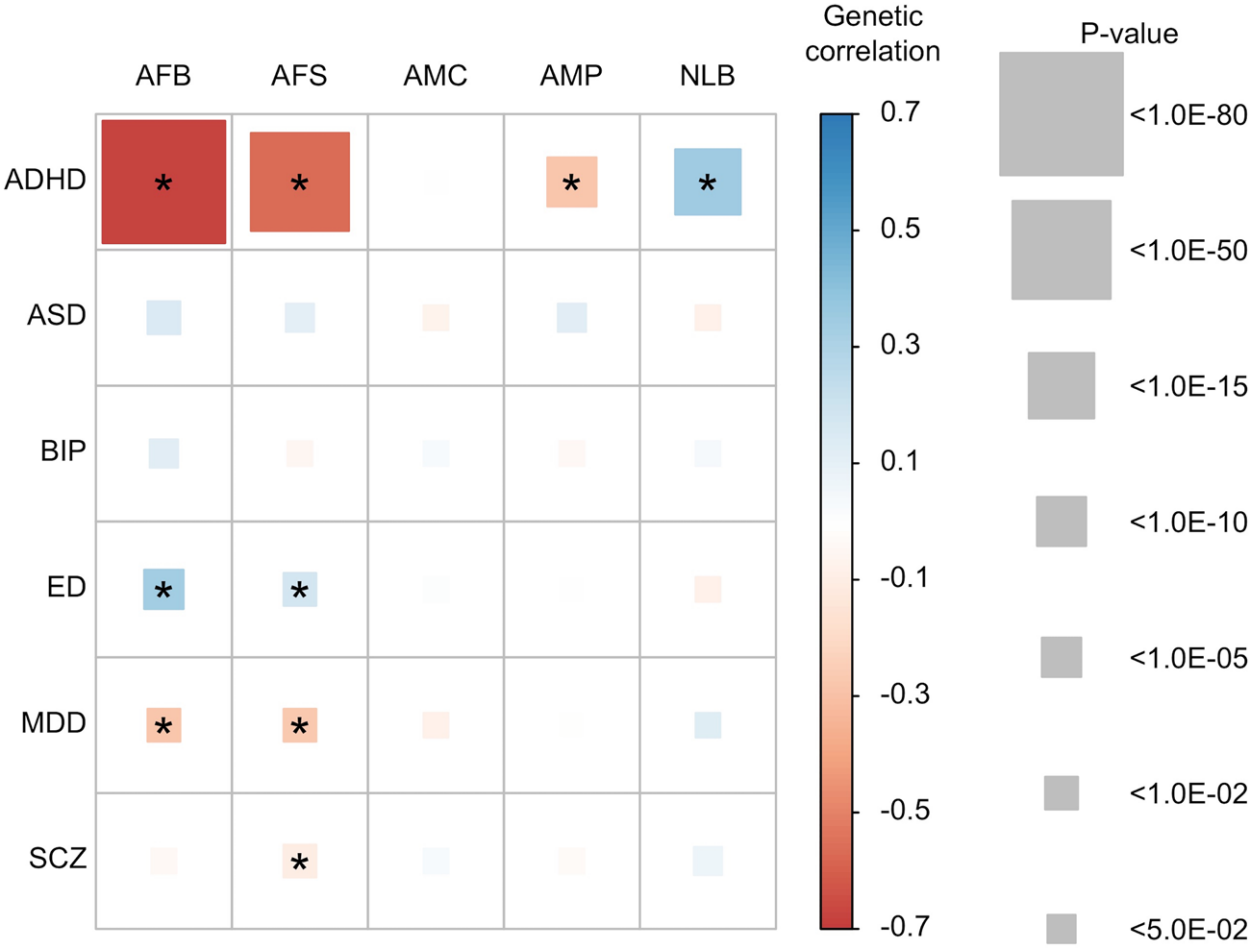
Genetic correlations between the five reproductive traits and the six psychiatric disorders estimated using the base model. Color of each box represents the level of estimated genetic correlation (blue for positive and red for negative correlation), and the size of squares represents its significance (p-value). Estimated genetic correlations that are significantly different from zero after Bonferroni correction (0.05/30) are marked with an asterisk. AFB: Age at first birth. AFS: Age at first sexual intercourse. AMC: Age at menarche. AMP: age at menopause. NLB: Number of live births. ADHD: Attention-Deficit/Hyperactivity Disorder. ASD: Autism spectrum disorder. ED: Eating disorder. BIP: Bipolar disorder. MDD: Major depressive disorder. SCZ: Schizophrenia. In the base model, the reproductive traits were adjusted for age at interview, year of birth, study center, genotype batch, and the first 15 principal components.

Figure 8 shows the estimated genetic correlation from LDSC for each pair of five reproductive traits and six psychiatric disorders. The detailed genetic correlations and P-value are in Table S11. Nine pairs of genetic correlations out of 30 were significantly different from zero after Bonferroni correction. For AFB analyses, the estimated genetic correlations were greater than zero (positive association) between AFB and ED (0.349 ± 0.061), and lower than zero (negative association) between AFB and ADHD (−0.677 ± 0.034) and AFB and MDD (−0.273 ± 0.069). Similarly, AFS was inversely correlated with ADHD (−0.563 ± 0.034), MDD (−0.265 ± 0.066) and SCZ (−0.100 ± 0.030) and positively correlated with ED (0.189 ± 0.055). For AMC and AMP, there was no significant genetic correlation except that between AMP and ADHD (−0.272 ± 0.038). NLB showed positive genetic correlation with ADHD (0.356 ± 0.042) and was non-significant for other pairs of traits. These results agreed with the analyses of mean difference of PRS (Figs 1–5) and linear prediction above (Fig. 7).

In the analyses where dependent variables were further adjusted for education, income levels, smoking and alcohol consumption status, the estimated genetic correlations between reproductive traits and psychiatric disorders were not substantially changed (Fig. S3 and Table S11), compared to those depicted in Fig. 8 and Table S11.

### Analysis of overlapping samples between PGC and UK biobank data

The intercepts between the reproductive traits and six psychiatric disorders were not significantly different from zero, indicating little overlaps between UK biobank and PGC samples (Figs S4 and S5). The sole exception was that the intercept between AFB and ED was significantly lower than zero (−0.017 ± SE 0.006), which may be due to sampling errors or excessive heterogeneity^40,41^. Most of the intercepts from the cross-trait LDSC analyses of the five reproductive traits were significantly different from zero, as expected (Figs S6 and S7). We note that the intercepts of AMP-AMC and AMC-NLB were not different from zero, which was because of the small phenotypic correlation between these pairs of traits.

### Causal effects of psychiatric disorder PRS on female reproduction traits

In the analysis using IVW regression, we found weak evidence (not significant after multiple testing correction) for a causal relationship between ADHD and AFB (β = −0.521, 95% confidence interval: −0.969 to −0.072, p-value = 0.031), and between ADHD and AFS (β = −0.407, 95% CI: −0.667 to −0.147, p-value = 0.010). None of the sensitivity analyses confirmed the significance except the weighted median approach, i.e. p-value = 0.042 for the causal relationship between ADHD and AFB and p-value = 0.016 for that between ADHD and AFS (which was also not significant after multiple testing correction) (Table 2). It was noted that the MR-Egger intercept estimate for the causal relationship between ADHD and AFS was significantly different from zero (p-value = 0.041), indicating that the signal of causal relationship might be due to pleiotropic effects. There was no notable evidence for causal relationship between any pair of psychiatric disorder PRS and reproduction traits in the analyses. The *I*^2^ statistics^48^ of MR-Egger were 0.96, 0.69 and 0.97 for ADHD, BIP and SCZ, mostly satisfying the ‘no measurement error’ assumption^48^ except BIP that had a weaker association signal than ADHD or SCZ (Figs 7 and 8).

**Table 2:** Two sample MR of causal association of psychiatric disorders on female reproduction traits of interest.

	Method	#Instruments	DF	AFB		AFS		AMC		AMP		NLB	
				Beta	p-value	Beta	p-value	Beta	p-value	Beta	p-value	Beta	p-value
ADHD	Inverse variance weighted	6	5	−0.521	0.031	−0.407	0.010	−0.029	0.553	−0.209	0.144	0.028	0.292
ADHD	MR Egger Intercept	6	4	−0.080	0.267	−0.073	0.041	−0.025	0.161	−0.044	0.393	−0.004	0.679
ADHD	MR Egger	6	4	0.290	0.679	0.336	0.264	0.223	0.215	0.236	0.649	0.071	0.521
ADHD	Weighted median	6	5	−0.414	0.042	−0.352	0.016	−0.038	0.371	−0.244	0.175	0.038	0.220
ADHD	Weighted mode	6	5	−0.429	0.143	−0.309	0.114	−0.040	0.461	−0.250	0.305	0.067	0.194
BIP	Inverse variance weighted	4	3	0.083	0.297	0.087	0.246	−0.029	0.493	−0.137	0.197	−0.001	0.933
BIP	MR Egger Intercept	4	2	0.008	0.881	0.048	0.272	−0.026	0.359	−0.069	0.318	0.002	0.846
BIP	MR Egger	4	2	0.035	0.918	−0.198	0.420	0.125	0.452	0.272	0.486	−0.013	0.836
BIP	Weighted median	4	3	0.090	0.324	0.052	0.408	0.003	0.916	−0.177	0.176	0.003	0.862
BIP	Weighted mode	4	3	0.147	0.305	0.044	0.615	0.015	0.649	−0.210	0.242	0.005	0.839
SCZ	Inverse variance weighted	76	75	−0.056	0.268	−0.062	0.061	0.024	0.208	0.001	0.974	0.006	0.424
SCZ	MR Egger Intercept	76	74	−0.003	0.842	−0.006	0.584	0.002	0.811	0.008	0.569	0.003	0.275
SCZ	MR Egger	76	74	−0.011	0.960	0.018	0.907	0.004	0.965	−0.107	0.583	−0.032	0.374
SCZ	Weighted median	76	75	0.011	0.830	−0.066	0.055	0.010	0.516	0.012	0.828	0.014	0.166
SCZ	Weighted mode	76	75	0.091	0.449	−0.112	0.295	−0.015	0.713	−0.008	0.953	0.026	0.333

AFB: Age at first birth. AFS: Age at first sexual intercourse. AMC: Age at menarche. AMP: age at menopause. NLB: Number of live births.

ADHD: Attention-Deficit/Hyperactivity Disorder. ASD: Autism spectrum disorder. ED: Eating disorder. BIP: Bipolar disorder. MDD: Major depressive disorder. SCZ: Schizophrenia.

## Discussion

We revealed the complex psychosocial genetic risk architecture underpinning the association between reproductive traits and six psychiatric disorders using the HapMap3 SNPs that are known to be reliable^1,37,38,40^. The strong genetic associations between ADHD and most reproductive traits (except AMC) were supported by three different approaches, the analyses of mean difference of PRS across five different (age) categories, linear prediction and genetic correlation using LDSC. Genetic associations between two of the reproductive traits (AFB and AFS) and both MDD and ED were consistently significant across the various analyses. The association between SCZ and AFB (and NLB) was significant in the analyses of mean difference of PRS and linear prediction although the estimated genetic correlation from LDSC analysis was not significant.

It is known that modelling genetic correlation between traits can increase accuracy significantly in predicting individual genetic risk for the traits^49,50^. Our findings of significant associations between female reproductive traits and between these traits and psychiatric disorders may be useful in improving female reproductive health, hence better child outcomes. For example, the high-risk group for ADHD could be informed about features of ADHD, such as impulsive behaviour and possible consequences of impulsivity. This intervention may lead to prevent them giving birth at an immature age, which can improve their reproductive health and the maternal environment for their baby. Furthermore, this information of genetic predisposition, combined with information on psychosocial factors, can be recorded as a form of family medical history, and used to monitor health of offspring.

Psychiatric disorders are highly heritable traits (e.g. ADHD or SCZ) while they persist in the population in a stable prevalence rate. It is questioned why natural selection has not excluded the causal mutations underlying psychiatric disorders. Three plausible hypotheses have been proposed. First, the impact of natural selection on the removal of existing causal mutations may be slower than the addition of new genetic mutations causing, for example, ADHD or SCZ. Second, pre-existing neutral mutations may interact with environments that have been new exposures for the population, and cause the disorders^51^. Third, causal mutations underlying psychiatric disorders have positive effects on reproductive success. The last hypothesis can be supported by our observation that NLB and ADHD have a strong positive genetic correlation (Figs 7 and 8). For SCZ and MDD, there is a suggestive signal for a positive genetic association with NLB (Figs 7 and 8), which agrees with a recent comprehensive study for SCZ^52^. However, this hypothesis is not supported by the results for ASD, BIP and ED.

ADHD is shown to be genetically associated with a set of reproductive traits such as AFB or AFS. However, we found no clear evidence that higher genetic risk of ADHD decreased AFB or AFS, from the MR analyses. The negative association between ADHD PRS and AFB or between ADHD PRS and AFS found in linear prediction and genetic correlation might be mostly because of pleiotropic effects. MR-Egger result also showed that pleiotropic effects explained the relationship between ADHD PRS and AFS. We did not find any evidence to support a causal relationship for any pair of psychiatric disorder PRS and reproduction traits, which agree with a recent study^53^ reporting that there is no evidence of a causal effect of genetic liability for schizophrenia on NLB or AFB. It was noted that we used a small number of SNPs as instruments in the MR analyses especially for ADHD and BIP. Therefore, a further study with a large number of instruments should be warranted when more genome-wide SNPs become available.

We report a novel shared genetic architecture between female reproductive traits, i.e. including strong positive genetic correlation between AFB and AFS, high negative genetic correlation between AFB and NLB, and AFS and NLB, moderate positive genetic correlation between AMP and AFB (AFS), moderate negative genetic correlation between AMP and NLB, and negligible genetic correlation between AMC and other reproductive traits. These reported relationships among the reproductive traits may help shed light on how causal genetic variants are involved and shared in the regulation of reproductive mechanisms at the genome-wide level. The strong positive and negative associations between AFB and AFS, and AFB and NLB are intuitive and well expected. However, other relationships (e.g. moderate positive association between AFB and AMP and negligible association between AMC and AMP) should be further investigated in a future study to identify the exact mechanism of menarche and menopause in relation to reproductive success^54^.

There are a number of limitations in this study. Firstly, we used the PRS or LDSC approach using GWAS summary statistics instead of individual-level genotype data for psychiatric disorders, for which we do not have access permissions. The power and accuracy of detection and estimation in the analyses could be increased when using individual-level genotype data^55^. Secondly, we tested causal relationship between the PRS of ADHD, BIP and SCZ and five reproductive traits in women and found no evidence of causality. A relatively small number of instruments might result in lack of power to detect such causal relationship if there is any. Thirdly, we only focused on reproductive traits in women because data on related male traits (e.g. AFB, AFS) are not fully available in the UKB. The collection of data on reproductive traits such as AFB in men, that would enable the study of genetic associations between psychiatric disorders and these reproductive traits should be a priority for the field. Fourthly, for the female reproductive traits, we used self-reported data and ascertained sample that is less representative of the general population. A fifth limitation is the possible confounding from factors such as a history of early childhood adversity and socioeconomic status (SES). A history of childhood adversity has been associated with a range of adverse mental health outcomes, including psychosis^56^, BIP^57^, and MDD^58^. With regard to SES, children living in low-income households have been found to have an increased risk for ADHD^59^. Low access to economic resources have been associated both with an earlier age of menarche, and in both females and males, a larger number of offspring at an earlier age^60^. In particular, a strong link has been observed between lower SES during childhood and parenthood during adolescence^61^. Therefore, in sensitivity analyses, we repeated the prediction using income level (as a measure of SES) as an additional variable.

In conclusion, we revealed the latent genetic architecture between the five reproductive traits in women and six psychiatric disorders. In particular, ADHD PRS and a set of reproductive traits were substantially associated through common genetic factors. There were also a number of robust associations between reproductive traits and ED, SCZ and MDD PRS. There was no evidence that the risk of any psychiatric disorder modulated the phenotypes of the female reproductive traits. Therefore, the associations found in this study were mostly due to genetic pleiotropic effects. Our findings can have potential to help improve reproductive health in women and their child outcomes. These findings also can help address an evolutionary hypothesis that causal mutations underlying ADHD, MDD and SCZ have positive effects on reproductive success.

### URLS

PGC: http://www.med.unc.edu/pgc/.

MTG2: https://sites.google.com/site/honglee0707/mtg2.

LDSC: https://github.com/bulik/ldsc.

## Supplementary information

Supplementary file

## Data Availability

The genome-wide analysis summary statistics of Psychiatric genomics consortium results can be downloaded from the public domain (http://www.med.unc.edu/pgc/). The individual genotype data of UK Biobank sample can be available via application to UK Biobank (http://www.ukbiobank.ac.uk/register-apply/).
