# Supplementary material for: The genetic relationship between female reproductive traits and six psychiatric disorders
Source: Sci Rep. 2019 Aug 19;9:12041. doi: 10.1038/s41598-019-48403-x (PMC6700195; doi:10.1038/s41598-019-48403-x)
Supplement: Supplementary file 1 — Supplementary file [file 41598_2019_48403_MOESM1_ESM.docx]

**The genetic relationship between female reproductive traits and six psychiatric disorders**

Guiyan Ni, Azmeraw Amare, Xuan Zhou, Natalie Mills, Jacob Gratten, and S. Hong Lee


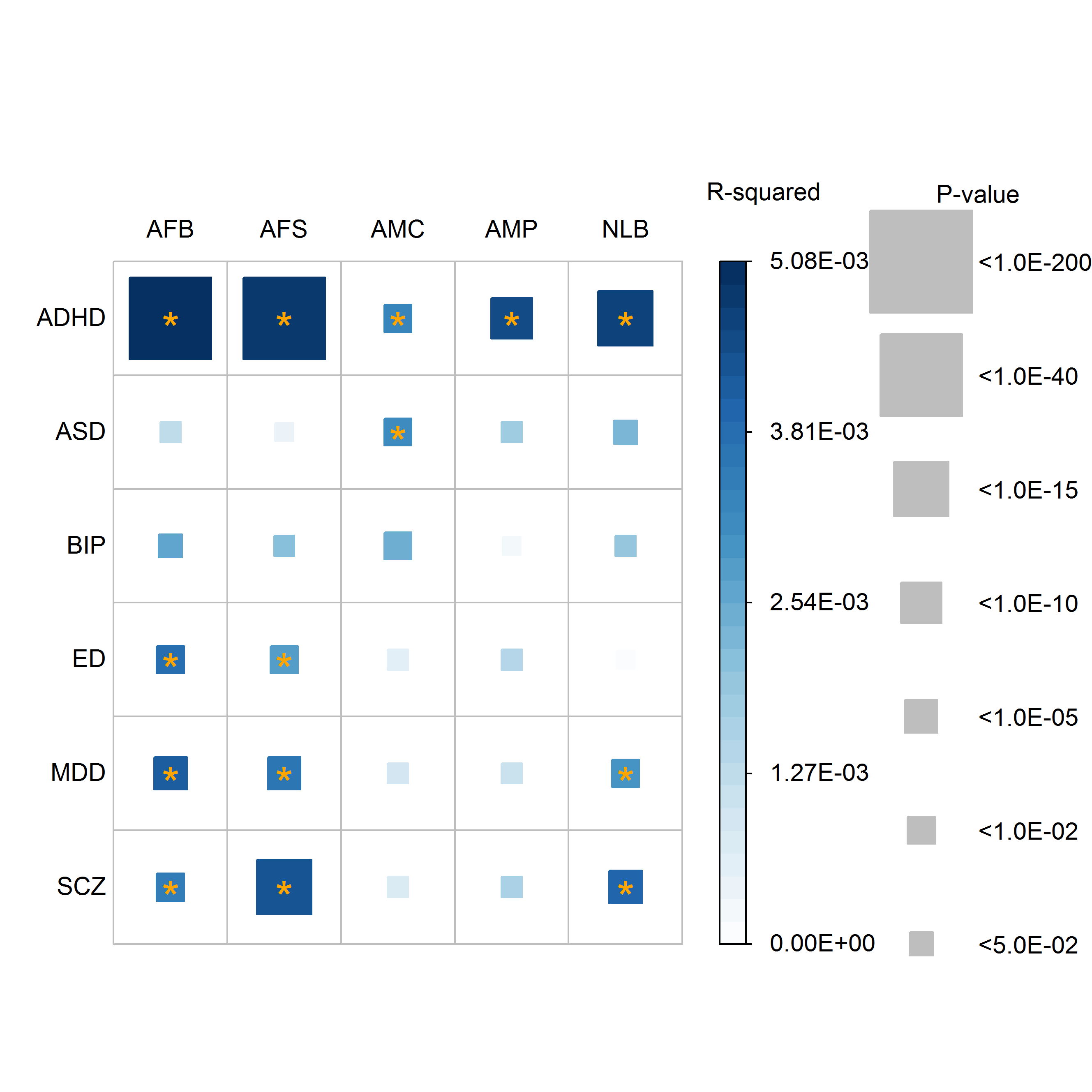


## Figure S1. Coefficient of determination (R^2^) and p-values for its significance based on a linear prediction model.

Color of each box represents the level of R-squared, and the size of squares represents its significance (p-value). R-squared that are significantly different from zero after Bonferroni correction (0.05/30) are marked with an asterisk.

Dependent variables were adjusted for age at interview, year of birth, assessment centre at which the participant consented, genotype batch, and the first 15 principal components, educational and income level, and smoking and alcohol consumption status.


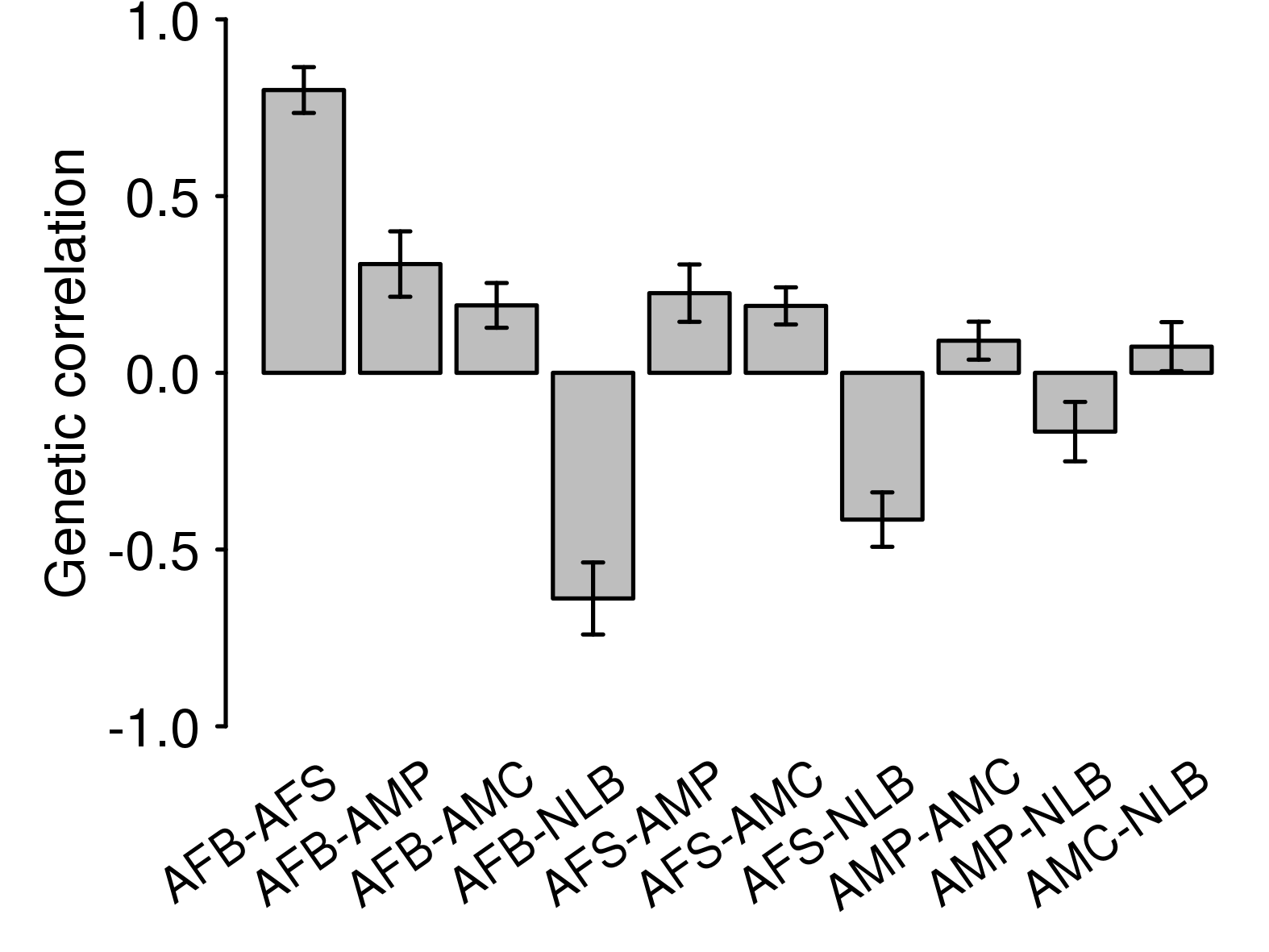


## Figure S2. Genetic correlations among the five reproductive traits estimated using the base model with additional adjustment for educational and income level, and smoking and alcohol consumption status.

In the base model, the reproductive traits were adjusted for age at interview, year of birth, study centre, genotype batch, and the first 15 principal components. Error Bars are 95% confidence intervals.

AFB: Age at first birth. AFS: Age at first sexual intercourse. AMC: Age at menarche. AMP: age at menopause. NLB: Number of live births


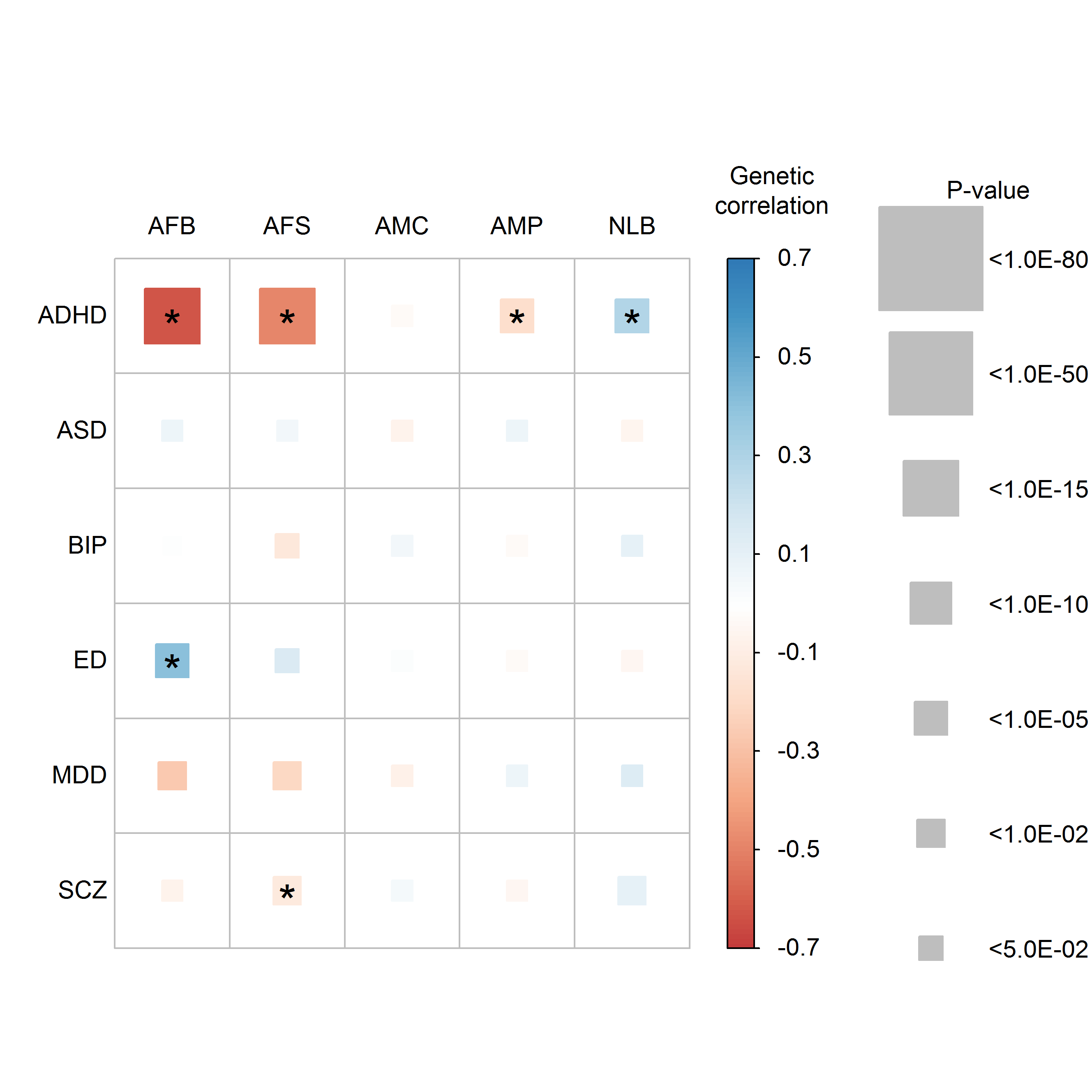


## Figure S3. Genetic correlations between the five female reproductive traits and the six psychiatric disorders estimated using the base model with additional adjustment for educational and income level, and smoking and alcohol consumption status.

Color of each box represents the level of estimated genetic correlation (blue for positive and red for negative correlation), and the size of squares represents its significance (p-value). Estimated genetic correlations that are significantly different from zero after Bonferroni correction (0.05/30) are marked with an asterisk.

AFB: Age at first birth. AFS: Age at first sexual intercourse. AMC: Age at menarche. AMP: age at menopause. NLB: Number of live births. ADHD: Attention-Deficit/Hyperactivity Disorder. ASD: Autism spectrum disorder. ED: Eating disorder. BIP: Bipolar disorder. MDD: Major depressive disorder. SCZ: Schizophrenia

In the base model, the reproductive traits were adjusted for age at interview, year of birth, study centre, genotype batch, and the first 15 principal components.


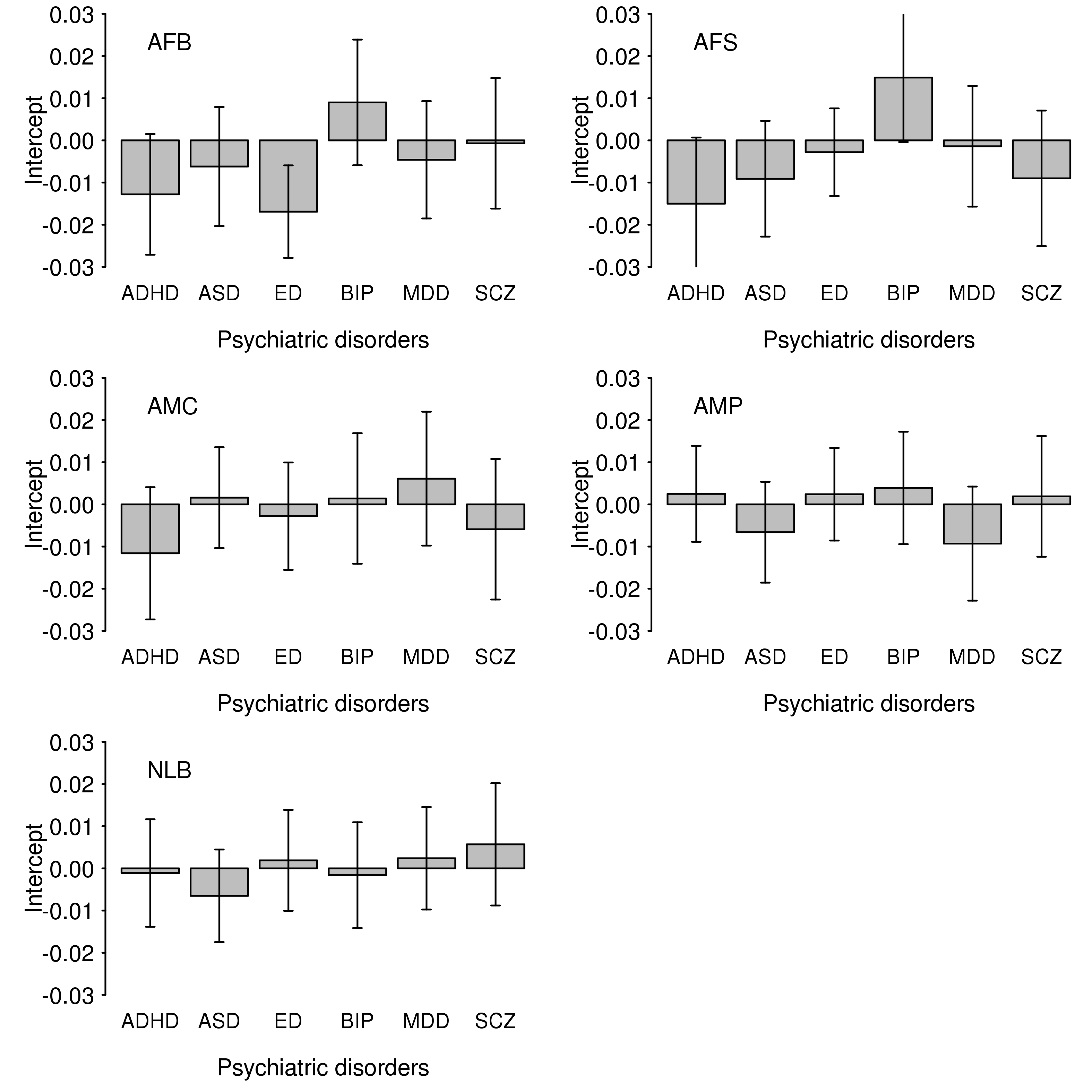


**Figure S4. Estimated intercepts from LDSC genetic correlation analyses based on the base model.**

AFB: Age at first birth. AFS: Age at first sexual intercourse. AMC: Age at menarche. AMP: age at menopause. NLB: Number of live births. ADHD: Attention-Deficit/Hyperactivity Disorder. ASD: Autism spectrum disorder. ED: Eating disorder. BIP: Bipolar disorder. MDD: Major depressive disorder. SCZ: Schizophrenia


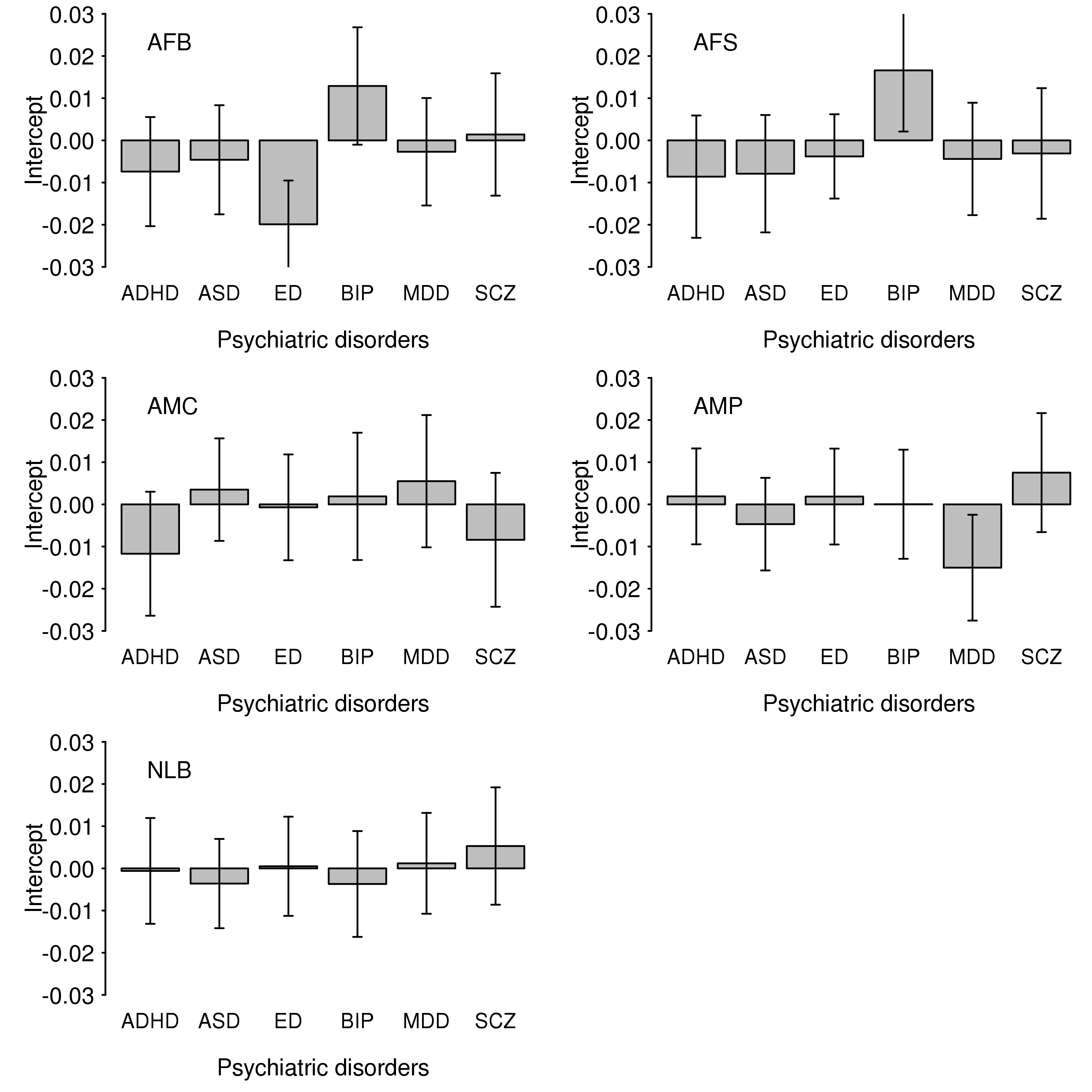


## Figure S5. Estimated intercepts from LDSC genetic correlation analyses based on the base model with additional adjustment for educational and income level, and smoking and alcohol consumption status.

AFB: Age at first birth. AFS: Age at first sexual intercourse. AMC: Age at menarche. AMP: age at menopause. NLB: Number of live births. ADHD: Attention-Deficit/Hyperactivity Disorder. ASD: Autism spectrum disorder. ED: Eating disorder. BIP: Bipolar disorder. MDD: Major depressive disorder. SCZ: Schizophrenia


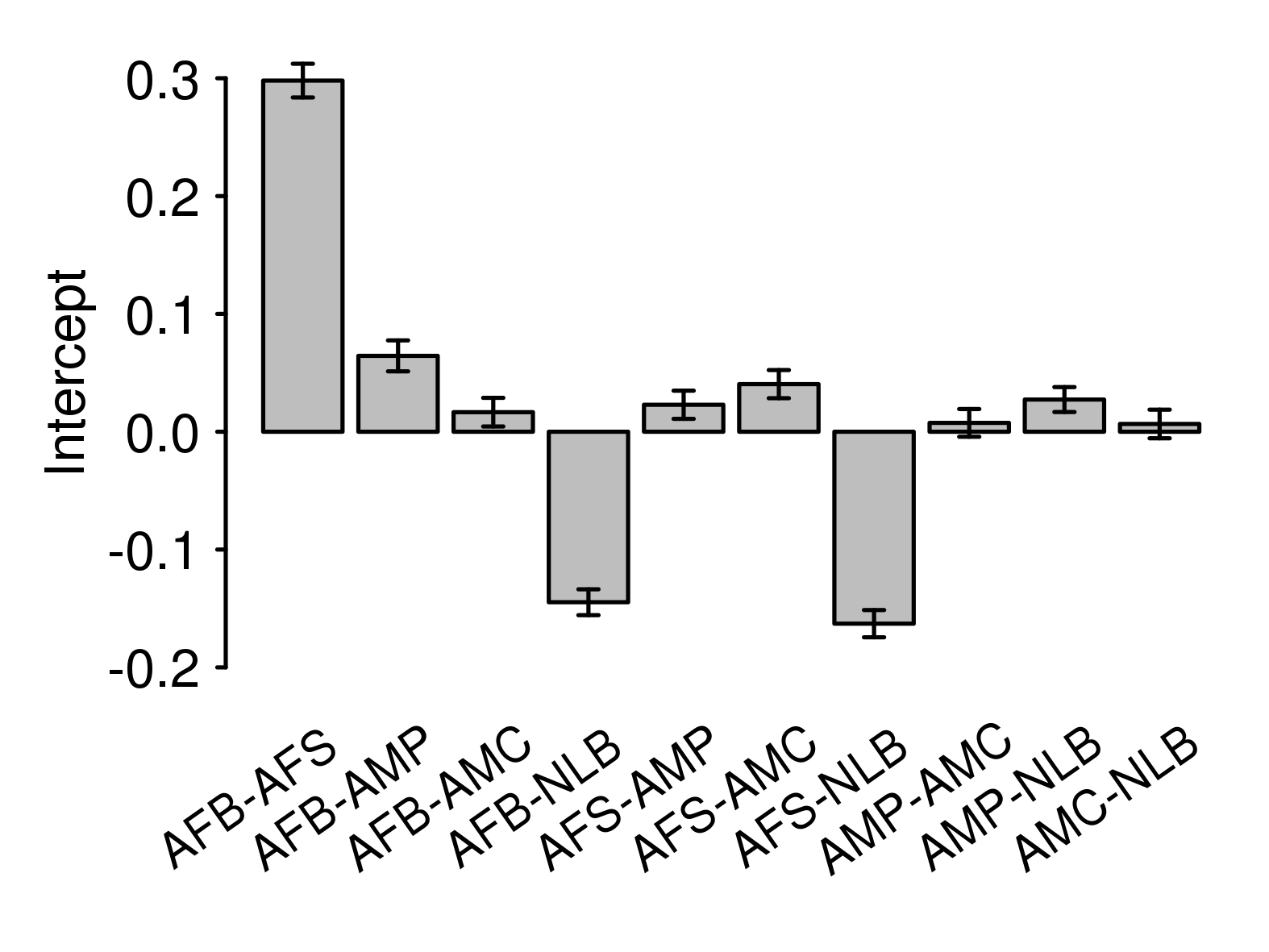


## Figure S6. Estimated intercepts from LDSC genetic correlation analyses for the five female reproductive traits based on the base model.

Bars are 95% confidence intervals.


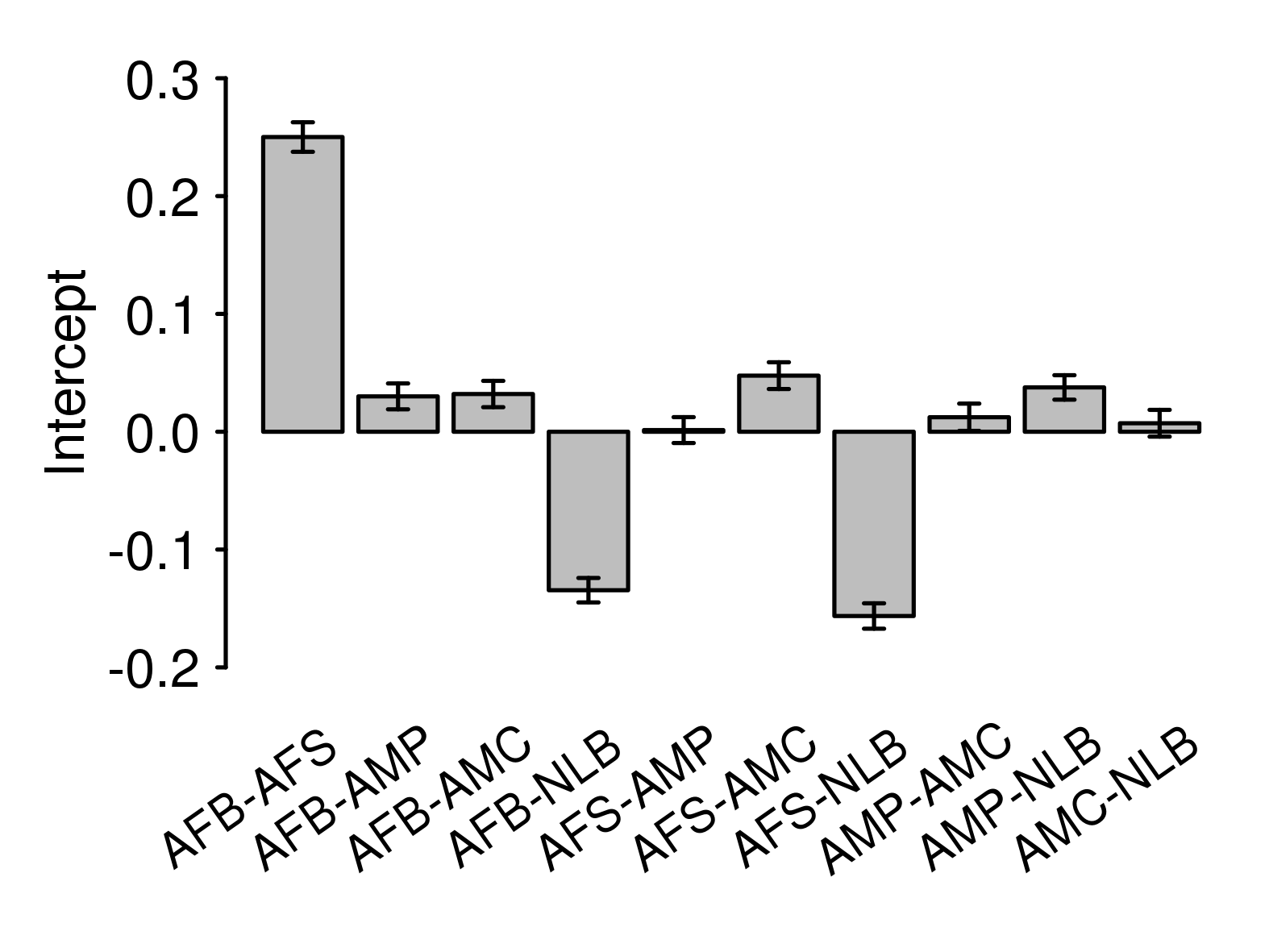


## Figure S7. Estimated intercepts from LDSC genetic correlation analyses among five female reproductive traits based on the base model with additional adjustment for educational and income level, and smoking and alcohol consumption status.

Bars are 95% confidence intervals.

## Table S1. The number of cases and controls used in the GWAS study for each of the six psychiatry disorders

|  | Case | Control | Total |
| --- | --- | --- | --- |
| ADHD | 20,183 | 35,191 | 55374 |
| ASD | 16,539 | 157,234 | 173773 |
| ED | 3,495 | 10,982 | 14477 |
| BIP | 7,481 | 9,250 | 16731 |
| MDD | 9,240 | 9,519 | 18759 |
| SCZ | 36,989 | 113,075 | 150064 |

## Table S2. P-values of pairwise comparisons between AFB groups on PRS of the six disorders.

|  | ADHD |  |  |  |  |
| --- | --- | --- | --- | --- | --- |
| ASD | AFB <20 | 20 ≤ AFB <25 | 25≤ AFB <30 | 30≤ AFB <35 | AFB ≥35 |
| AFB <20 | - | **5.7E-44** | **9.1E-179** | **2.0E-184** | **1.3E-100** |
| 20≤ AFB <25 | 6.9E-01 | - | **3.7E-116** | **3.5E-113** | **3.0E-49** |
| 25≤ AFB <30 | 2.5E-02 | **3.6E-05** | - | **1.4E-09** | **2.1E-07** |
| 30≤ AFB <35 | 4.2E-03 | **1.9E-05** | 2.1E-01 | - | 1.12E-01 |
| AFB ≥35 | 2.2E-01 | 1.0E-01 | 9.5E-01 | 4.7E-01 | - |
|  | ED |  |  |  |  |
| BIP | AFB <20 | 20 ≤ AFB <25 | 25≤ AFB <30 | 30≤ AFB <35 | AFB ≥35 |
| AFB <20 | - | 7.1E-02 | **1.5E-06** | **2.8E-09** | **1.9E-06** |
| 20≤ AFB <25 | 6.2E-01 | - | **3.8E-06** | **6.6E-09** | **3.6E-05** |
| 25≤ AFB <30 | 5.6E-02 | 1.0E-04 | - | 1.6E-02 | 2.5E-02 |
| 30≤ AFB <35 | 8.4E-03 | **3.9E-05** | 1.8E-01 | - | 3.9E-01 |
| AFB ≥35 | 5.0E-04 | **2.2E-05** | 7.7E-03 | 7.7E-02 | - |
|  | MDD |  |  |  |  |
| SCZ | AFB <20 | 20 ≤ AFB <25 | 25≤ AFB <30 | 30≤ AFB <35 | AFB ≥35 |
| AFB <20 | - | **6.3E-05** | **8.8E-14** | **1.9E-14** | **5.1E-05** |
| 20≤ AFB <25 | **1.1E-08** | - | **1.2E-07** | **3.1E-08** | 6.0E-02 |
| 25≤ AFB <30 | **2.9E-19** | **5.9E-07** | - | 9.8E-02 | 7.3E-01 |
| 30≤ AFB <35 | **1.1E-09** | 1.4E-01 | 2.5E-02 | - | 2.3E-01 |
| AFB ≥35 | 4.6E-03 | 5.2E-01 | 5.6E-03 | 1.6E-01 | - |

AFB: Age at first birth. ADHD: Attention-Deficit/Hyperactivity Disorder. ASD: Autism spectrum disorder. ED: Eating disorder. BIP: Bipolar disorder. MDD: Major depressive disorder. SCZ: Schizophrenia

Considering the 10 pairwise comparisons of age categories in each combination of reproductive trait and psychiatric disorder, the significance threshold is 0.05 / (10×5×6) = 1.7E-04. The comparisons that passed the significance threshold were highlighted in bold.

## Table S3. P-value of pairwise comparisons between AFS groups on PRS of the six disorders.

|  | ADHD |  |  |  |  |
| --- | --- | --- | --- | --- | --- |
| ASD | AFS<16 | 16≤AFS<20 | 20≤AFS<24 | 24≤AFS<28 | AFS≥28 |
| AFS<16 | - | **3.9E-11** | **7.7E-87** | **3.0E-177** | **8.2E-49** |
| 16≤AFS<20 | 9.2E-01 | - | **1.0E-83** | **3.2E-192** | **1.8E-36** |
| 20≤AFS<24 | 2.2E-01 | 4.2E-02 | - | **1.1E-58** | **1.6E-14** |
| 24≤AFS<28 | 2.6E-02 | 2.0E-03 | 9.5E-02 | - | 1.6E-02 |
| AFS≥28 | 1.4E-02 | 8.7E-03 | 3.5E-02 | 1.3E-01 | - |
|  | ED |  |  |  |  |
| BIP | AFS<16 | 16≤AFS<20 | 20≤AFS<24 | 24≤AFS<28 | AFS≥28 |
| AFS<16 | - | 4.1E-02 | 9.8E-02 | 1.2E-02 | 5.6E-02 |
| 16≤AFS<20 | 8.4E-01 | - | **7.1E-09** | **6.1E-09** | 4.3E-03 |
| 20≤AFS<24 | 1.4E-01 | 1.1E-02 | - | 1.2E-01 | 1.8E-01 |
| 24≤AFS<28 | 2.4E-02 | 1.1E-03 | 1.6E-01 | - | 4.1E-01 |
| AFS≥28 | 5.1E-01 | 4.4E-01 | 9.3E-01 | 7.1E-01 | - |
|  | MDD |  |  |  |  |
| SCZ | AFS<16 | 16≤AFS<20 | 20≤AFS<24 | 24≤AFS<28 | AFS≥28 |
| AFS<16 | - | 3.0E-01 | 1.2E-02 | 2.0E-04 | 1.3E-01 |
| 16≤AFS<20 | 9.0E-04 | - | 3.2E-02 | 3.0E-04 | 2.6E-01 |
| 20≤AFS<24 | **3.8E-14** | 9.1E-10 | - | 3.1E-02 | 5.7E-01 |
| 24≤AFS<28 | **1.5E-13** | **1.1E-08** | 2.7E-01 | - | 9.0E-01 |
| AFS≥28 | **4.8E-07** | **1.0E-04** | 2.3E-02 | 6.1E-02 | - |

AFS: Age at first sexual intercourse. ADHD: Attention-Deficit/Hyperactivity Disorder. ASD: Autism spectrum disorder. ED: Eating disorder. BIP: Bipolar disorder. MDD: Major depressive disorder. SCZ: Schizophrenia

Considering the 10 pairwise comparisons of age categories in each combination of reproductive trait and psychiatric disorder, the significance threshold is 0.05 / (10×5×6) = 1.7E-04. The comparisons that passed the significance threshold were highlighted in bold.

## Table S4. P-value of pairwise comparisons between AMC groups on PRS of the six disorders.

|  | ADHD |  |  |  |  |
| --- | --- | --- | --- | --- | --- |
| ASD | AMC<11 | 11≤AMC<13 | 13≤AMC<15 | 15≤AMC<17 | AMC≥17 |
| AMC<11 | - | **1.2E-12** | **1.2E-18** | **5.1E-07** | 2.0E-01 |
| 11≤AMC<13 | 7.6E-02 | - | 5.0E-04 | 7.2E-03 | 1.4E-02 |
| 13≤AMC<15 | 3.5E-01 | 6.3E-02 | - | **6.5E-08** | 8.0E-04 |
| 15≤AMC<17 | 3.3E-02 | **6.9E-11** | **1.0E-07** | - | 1.3E-01 |
| AMC≥17 | 5.4E-01 | 7.6E-01 | 8.6E-01 | 5.5E-02 | - |
|  | ED |  |  |  |  |
| BIP | AMC<11 | 11≤AMC<13 | 13≤AMC<15 | 15≤AMC<17 | AMC≥17 |
| AMC<11 | - | 2.5E-02 | 8.4E-03 | 3.1E-01 | 5.3E-01 |
| 11≤AMC<13 | 3.0E-01 | - | 4.5E-01 | 6.8E-02 | 5.8E-01 |
| 13≤AMC<15 | 7.4E-01 | 3.2E-03 | - | 1.4E-02 | 4.6E-01 |
| 15≤AMC<17 | 8.4E-01 | 4.7E-02 | 8.6E-01 | - | 9.2E-01 |
| AMC≥17 | 8.2E-01 | 4.1E-01 | 9.4E-01 | 9.0E-01 | - |
|  | MDD |  |  |  |  |
| SCZ | AMC<11 | 11≤AMC<13 | 13≤AMC<15 | 15≤AMC<17 | AMC≥17 |
| AMC<11 | - | 7.5E-01 | 4.6E-01 | 7.3E-01 | 1.4E-01 |
| 11≤AMC<13 | 5.4E-01 | - | 2.4E-02 | 2.8E-01 | 1.5E-01 |
| 13≤AMC<15 | 9.6E-02 | 2.5E-02 | - | 5.6E-01 | 4.1E-02 |
| 15≤AMC<17 | 1.1E-01 | 7.2E-02 | 8.7E-01 | - | 7.4E-02 |
| AMC≥17 | 3.4E-01 | 4.6E-01 | 8.6E-01 | 9.0E-01 | - |

AMC: Age at menarche. ADHD: Attention-Deficit/Hyperactivity Disorder. ASD: Autism spectrum disorder. ED: Eating disorder. BIP: Bipolar disorder. MDD: Major depressive disorder. SCZ: Schizophrenia

Considering the 10 pairwise comparisons of age categories in each combination of reproductive trait and psychiatric disorder, the significance threshold is 0.05 / (10×5×6) = 1.7E-04. The comparisons that passed the significance threshold were highlighted in bold.

## Table S5. P-values of pairwise comparisons between AMP groups on PRS of the six disorders.

|  | ADHD |  |  |  |  |
| --- | --- | --- | --- | --- | --- |
| ASD | AMP<40 | 40≤AMP<46) | 46≤AMP<52 | 52≤AMP<58 | AMP≥58 |
| AMP<40 | - | 1.0E-03 | **8.9E-14** | **7.0E-21** | **1.0E-16** |
| 40≤AMP<46 | 4.7E-02 | - | **8.8E-14** | **2.7E-31** | **3.0E-18** |
| 46≤AMP<52 | 2.6E-01 | 7.7E-02 | - | **2.9E-06** | 2.9E-02 |
| 52≤AMP<58 | 2.2E-02 | 7.8E-01 | 2.7E-03 | - | 1.1E-01 |
| AMP≥58 | 1.9E-02 | 6.0E-01 | 8.4E-03 | 7.0E-01 | - |
|  | ED |  |  |  |  |
| BIP | AMP<40 | 40≤AMP<46) | 46≤AMP<52 | 52≤AMP<58 | AMP≥58 |
| AMP<40 | - | 7.8E-01 | 8.0E-01 | 9.2E-01 | 2.9E-01 |
| 40≤AMP<46 | 5.4E-02 | - | 9.6E-01 | 7.0E-01 | 2.2E-02 |
| 46≤AMP<52 | 1.5E-01 | 2.7E-01 | - | 6.6E-01 | 6.2E-03 |
| 52≤AMP<58 | 1.5E-01 | 2.2E-01 | 9.6E-01 | - | 7.1E-03 |
| AMP≥58 | 8.1E-01 | **1.0E-04** | 6.0E-04 | 2.0E-04 | - |
|  | MDD |  |  |  |  |
| SCZ | AMP<40 | 40≤AMP<46) | 46≤AMP<52 | 52≤AMP<58 | AMP≥58 |
| AMP<40 | - | 9.7E-01 | 1.9E-01 | 4.0E-02 | 6.9E-02 |
| 40≤AMP<46 | 1.6E-01 | - | 1.2E-02 | 0.0E+00 | 1.1E-03 |
| 46≤AMP<52 | 1.6E-01 | 9.2E-01 | - | 6.4E-02 | 2.6E-01 |
| 52≤AMP<58 | 8.3E-01 | 1.2E-02 | 1.2E-03 | - | 7.4E-01 |
| AMP≥58 | 6.0E-01 | 7.0E-04 | 1.0E-04 | 8.7E-02 | - |

AMP: age at menopause. ADHD: Attention-Deficit/Hyperactivity Disorder. ASD: Autism spectrum disorder. ED: Eating disorder. BIP: Bipolar disorder. MDD: Major depressive disorder. SCZ: Schizophrenia

Considering the 10 pairwise comparisons of age categories in each combination of reproductive trait and psychiatric disorder, the significance threshold is 0.05 / (10×5×6) = 1.7E-04. The comparisons that passed the significance threshold were highlighted in bold.

## Table S6. P-values of pairwise comparisons between NLB groups on PRS of the six disorders.

|  | ADHD |  |  |  |  |
| --- | --- | --- | --- | --- | --- |
| ASD | NLB=0 | NLB=1 | NLB=2 | NLB=3 | NLB>3 |
| NLB=0 | - | **8.6E-29** | **2.8E-17** | **7.8E-29** | **2.1E-52** |
| NLB=1 | 4.9E-02 | - | **8.4E-08** | 4.1E-01 | **1.8E-11** |
| NLB=2 | 9.2E-07 | 4.1E-02 | - | **9.5E-07** | **2.6E-29** |
| NLB=3 | 2.0E-04 | 1.6E-01 | 6.5E-01 | - | **3.3E-14** |
| NLB>3 | 7.4E-02 | 7.5E-01 | 2.9E-01 | 4.7E-01 | - |
|  | ED |  |  |  |  |
| BIP | NLB=0 | NLB=1 | NLB=2 | NLB=3 | NLB>3 |
| NLB=0 | - | 2.0E-01 | 2.2E-01 | 8.9E-01 | 1.1E-01 |
| NLB=1 | 9.2E-01 | - | 7.0E-01 | 1.6E-01 | 5.5E-01 |
| NLB=2 | 1.8E-01 | 2.0E-01 | - | 1.6E-01 | 3.4E-01 |
| NLB=3 | 3.9E-01 | 4.9E-01 | 2.0E-02 | - | 9.3E-02 |
| NLB>3 | 1.5E-02 | 2.4E-02 | 6.0E-04 | 6.7E-02 | - |
|  | MDD |  |  |  |  |
| SCZ | NLB=0 | NLB=1 | NLB=2 | NLB=3 | NLB>3 |
| NLB=0 | - | 2.3E-03 | 8.4E-01 | 2.0E-04 | **1.1E-06** |
| NLB=1 | **6.7E-11** | - | 2.0E-04 | 7.3E-01 | 1.3E-02 |
| NLB=2 | 6.2E-01 | **1.4E-12** | - | **5.5E-06** | **8.2E-08** |
| NLB=3 | **1.0E-05** | 1.5E-02 | **2.1E-06** | - | 2.0E-02 |
| NLB>3 | **7.7E-11** | 1.2E-01 | **2.1E-11** | 6.0E-04 | - |

NLB: Number of live births. ADHD: Attention-Deficit/Hyperactivity Disorder. ASD: Autism spectrum disorder. ED: Eating disorder. BIP: Bipolar disorder. MDD: Major depressive disorder. SCZ: Schizophrenia

Considering the 10 pairwise comparisons of age categories in each combination of reproductive trait and psychiatric disorder, the significance threshold is 0.05 / (10×5×6) = 1.7E-04. The comparisons that passed the significance threshold were highlighted in bold.

## Table S7. Coefficient of determination (R^2^) and p-values for its significance based on a linear prediction model

| **PRS** | **Y** | R^2^ | P | **Y** | R^2^ | P |
| --- | --- | --- | --- | --- | --- | --- |
| ADHD | **AFB** | 1.10E-02 | **1.20E-303** | **AFS** | 7.40E-03 | **3.30E-253** |
| ASD |  | 2.00E-04 | **7.60E-07** |  | 7.30E-05 | **7.10E-04** |
| ED |  | 4.60E-04 | **6.50E-14** |  | 1.90E-04 | **4.70E-08** |
| BIP |  | 3.20E-04 | **6.30E-10** |  | 5.50E-07 | 7.70E-01 |
| MDD |  | 5.90E-04 | **2.20E-17** |  | 3.60E-04 | **5.20E-14** |
| SCZ |  | 9.10E-05 | **8.90E-04** |  | 6.00E-04 | **3.50E-22** |
| ADHD | **AMC** | 2.40E-05 | 4.20E-02 | **AMP** | 1.30E-03 | **3.20E-31** |
| ASD |  | 1.20E-04 | **5.30E-06** |  | 8.50E-05 | 3.30E-03 |
| ED |  | 9.00E-08 | 9.00E-01 |  | 3.90E-07 | 8.40E-01 |
| BIP |  | 2.40E-05 | 4.20E-02 |  | 3.10E-10 | 1.00E+00 |
| MDD |  | 5.60E-06 | 3.30E-01 |  | 1.80E-05 | 1.70E-01 |
| SCZ |  | 3.80E-06 | 4.20E-01 |  | 2.70E-05 | 9.80E-02 |
| ADHD | **NLB** | 1.10E-03 | **9.70E-43** |  |  |  |
| ASD |  | 6.70E-05 | **5.70E-04** |  |  |  |
| ED |  | 6.10E-06 | 3.00E-01 |  |  |  |
| BIP |  | 6.70E-06 | 2.80E-01 |  |  |  |
| MDD |  | 8.20E-05 | **1.30E-04** |  |  |  |
| SCZ |  | 1.40E-04 | **7.20E-07** |  |  |  |

Dependent variables were adjusted for age at interview, year of birth, assessment centre at which the participant consented, genotype batch, and the first 15 principal components. The number of records used for the analyses was 121,544 for AFB, 156,143 for AFS, 102,386 for AMP, and 172,856 for AMC and 177,744 for NLB.

AFB: age at first firth. AFS: age first sexual intercourse. AMP: age at menopause. AMC: age at menarche. NLB: number of live births. ADHD: Attention-Deficit/Hyperactivity Disorder. ASD: Autism spectrum disorder. ED: Eating disorder. BIP: Bipolar disorder. MDD: Major depressive disorder. SCZ: Schizophrenia

Considering the number of reproductive traits and psychiatric disorders, the significance threshold is 0.05 / (5×6) = 1.7E-03. The results that passed the significance threshold were highlighted in bold.

## Table S8. Coefficient of determination (R^2^) and p-values for its significance based on a polynomial prediction model.

| **PRS** | **Y** | R^2^ | P-slope1 | P-slope2 | **PRS** | **Y** | R^2^ | P-slope1 | P-slope2 |
| --- | --- | --- | --- | --- | --- | --- | --- | --- | --- |
| ADHD | **AFB** | 1.10E-02 | **2.70E-303** | 4.20E-01 | ADHD | **AFS** | 7.40E-03 | **3.10E-253** | 1.00E-01 |
| ASD |  | 2.00E-04 | **7.40E-07** | 6.00E-01 | ASD |  | 8.30E-05 | **7.20E-04** | 2.20E-01 |
| ED |  | 4.80E-04 | **7.00E-14** | 1.30E-01 | ED |  | 2.30E-04 | **4.90E-08** | 1.60E-02 |
| BIP |  | 3.20E-04 | **6.30E-10** | 6.90E-01 | BIP |  | 7.50E-06 | 7.70E-01 | 3.00E-01 |
| MDD |  | 6.00E-04 | **2.10E-17** | 5.60E-01 | MDD |  | 3.70E-04 | **5.40E-14** | 2.20E-01 |
| SCZ |  | 1.30E-04 | **8.50E-04** | 2.60E-02 | SCZ |  | 6.00E-04 | **3.60E-22** | 5.20E-01 |
| ADHD | **AMC** | 3.80E-05 | 4.20E-02 | 1.30E-01 | ADHD | **AMP** | 1.30E-03 | **3.90E-31** | 6.70E-01 |
| ASD |  | 1.20E-04 | **5.20E-06** | 6.00E-01 | ASD |  | 8.50E-05 | 3.30E-03 | 7.80E-01 |
| ED |  | 4.50E-06 | 9.00E-01 | 3.80E-01 | ED |  | 7.50E-07 | 8.40E-01 | 8.50E-01 |
| BIP |  | 2.70E-05 | 4.20E-02 | 4.60E-01 | BIP |  | 2.70E-06 | 9.90E-01 | 6.00E-01 |
| MDD |  | 5.60E-06 | 3.30E-01 | 9.80E-01 | MDD |  | 1.90E-05 | 1.70E-01 | 7.60E-01 |
| SCZ |  | 9.60E-06 | 4.20E-01 | 3.20E-01 | SCZ |  | 3.00E-05 | 9.70E-02 | 5.80E-01 |
| ADHD | **NLB** | 1.10E-03 | **9.40E-43** | 3.30E-01 |  |  |  |  |  |
| ASD |  | 7.70E-05 | **5.70E-04** | 1.80E-01 |  |  |  |  |  |
| ED |  | 1.40E-05 | 3.00E-01 | 2.30E-01 |  |  |  |  |  |
| BIP |  | 7.50E-06 | 2.80E-01 | 7.10E-01 |  |  |  |  |  |
| MDD |  | 8.60E-05 | **1.30E-04** | 4.30E-01 |  |  |  |  |  |
| SCZ |  | 1.40E-04 | **7.20E-07** | 9.40E-01 |  |  |  |  |  |

The same data and pre-adjustment as in Table S1 were used. Dependent variables were adjusted for age at interview, year of birth, assessment centre at which the participant consented, genotype batch, the first 15 principal components. The number of records used for the analyses was 98975 for AFB, 133,657 for AFS, 82,873 for AMP, and 143,392 for AMC, and 146,802 for NLB. AFB: age at first firth. AFS: age first sexual intercourse. AMP: age at menopause. AMC: age at menarche. NLB: number of live births. ADHD: Attention-Deficit/Hyperactivity Disorder. ASD: Autism spectrum disorder. ED: Eating disorder. BIP: Bipolar disorder. MDD: Major depressive disorder. SCZ: Schizophrenia

P-slope1: p-value of testing the slope at the first degree of the polynomial function differ from zero.

P-slope2: p-value of testing the slope at the second degree of the polynomial function differ from zero

Considering the number of reproductive traits and psychiatric disorders, the significance threshold is 0.05 / (5×6) = 1.7E-03. The results that passed the significance threshold were highlighted in bold.

## Table S9. Coefficient of determination (R^2^) and p-values for its significance based on a linear and polynomial prediction models.

| **Y** | **PRS** | **Linear prediction model** | | **polynomial prediction model** | | |
| --- | --- | --- | --- | --- | --- | --- |
|  |  | **R^2^** | P | R^2^ | P-slope1 | P-slope2 |
| **AFB** | ADHD | 5.1E-03 | **1.0E-111** | 5.1E-03 | **1.2E-111** | 5.2E-01 |
|  | ASD | 1.1E-05 | 2.9E-01 | 4.0E-05 | 2.9E-01 | 9.3E-02 |
|  | ED | 1.9E-04 | **1.4E-05** | 2.0E-04 | **1.4E-05** | 4.9E-01 |
|  | BIP | 5.0E-05 | 2.6E-02 | 5.6E-05 | 2.6E-02 | 4.6E-01 |
|  | MDD | 2.5E-04 | **7.0E-07** | 2.5E-04 | **7.0E-07** | 8.6E-01 |
|  | SCZ | 1.3E-04 | **3.7E-04** | 1.7E-04 | **3.7E-04** | 4.6E-02 |
| **AFS** | ADHD | 4.0E-03 | **4.4E-118** | 4.0E-03 | **6.5E-118** | 5.7E-02 |
|  | ASD | 2.9E-06 | 5.4E-01 | 1.1E-05 | 5.4E-01 | 3.1E-01 |
|  | ED | 7.9E-05 | **1.1E-03** | 1.0E-04 | **1.2E-03** | 9.9E-02 |
|  | BIP | 2.6E-05 | 6.2E-02 | 3.7E-05 | 6.1E-02 | 2.2E-01 |
|  | MDD | 1.9E-04 | **5.0E-07** | 2.0E-04 | **5.1E-07** | 2.6E-01 |
|  | SCZ | 5.4E-04 | **1.9E-17** | 5.5E-04 | **1.9E-17** | 3.6E-01 |
| **AMC** | ADHD | 9.7E-05 | **2.0E-04** | 1.1E-04 | **2.1E-04** | 1.8E-01 |
|  | ASD | 9.3E-05 | **2.5E-04** | 9.3E-05 | **2.5E-04** | 9.7E-01 |
|  | ED | 3.7E-06 | 4.7E-01 | 1.6E-05 | 4.7E-01 | 1.9E-01 |
|  | BIP | 4.9E-05 | 8.0E-03 | 5.2E-05 | 8.0E-03 | 5.0E-01 |
|  | MDD | 5.2E-06 | 3.9E-01 | 5.3E-06 | 3.9E-01 | 8.8E-01 |
|  | SCZ | 4.4E-06 | 4.3E-01 | 5.1E-06 | 4.3E-01 | 7.6E-01 |
| **AMP** | ADHD | 6.2E-04 | **6.3E-13** | 6.3E-04 | **7.2E-13** | 7.2E-01 |
|  | ASD | 2.4E-05 | 1.6E-01 | 2.5E-05 | 1.6E-01 | 7.6E-01 |
|  | ED | 1.7E-05 | 2.3E-01 | 1.8E-05 | 2.4E-01 | 8.0E-01 |
|  | BIP | 2.0E-06 | 6.8E-01 | 8.8E-06 | 6.8E-01 | 4.5E-01 |
|  | MDD | 7.6E-06 | 4.3E-01 | 1.2E-05 | 4.3E-01 | 5.5E-01 |
|  | SCZ | 2.2E-05 | 1.8E-01 | 2.2E-05 | 1.8E-01 | 9.5E-01 |
| **NLB** | ADHD | 6.5E-04 | **1.9E-22** | 6.6E-04 | **1.6E-22** | 2.3E-01 |
|  | ASD | 3.2E-05 | 3.0E-02 | 5.2E-05 | 2.9E-02 | 8.7E-02 |
|  | ED | 1.6E-06 | 6.3E-01 | 6.3E-06 | 6.3E-01 | 4.1E-01 |
|  | BIP | 2.6E-05 | 5.1E-02 | 2.7E-05 | 5.1E-02 | 7.6E-01 |
|  | MDD | 8.3E-05 | **5.0E-04** | 9.5E-05 | **5.0E-04** | 1.8E-01 |
|  | SCZ | 2.2E-04 | **1.5E-08** | 2.2E-04 | **1.5E-08** | 9.2E-01 |

Dependent variables were adjusted for age at interview, year of birth, assessment centre at which the participant consented, genotype batch, the first 15 principal components, socioeconomic status, and smoking and alcohol drinking status. The number of records used for the analyses was 98975 for AFB, 133,657 for AFS, 82,873 for AMP, and 143,392 for AMC, and 146,802 for NLB.

AFB: age at first firth. AFS: age first sexual intercourse. AMP: age at menopause. AMC: age at menarche. NLB: number of live births. ADHD: Attention-Deficit/Hyperactivity Disorder. ASD: Autism spectrum disorder. ED: Eating disorder. BIP: Bipolar disorder. MDD: Major depressive disorder. SCZ: Schizophrenia

P-slope1: p-value of testing the slope at the first degree of the polynomial function differ from zero.

P-slope2: p-value of testing the slope at the second degree of the polynomial function differ from zero

Considering the number of reproductive traits and psychiatric disorders, the significance threshold is 0.05 / (5×6) = 1.7E-03. The results that passed the significance threshold were highlighted in bold.

## Table S10. Coefficient of determination (R2) and p-values for its significance based on a linear prediction model when PRS estimated from SNPs based on different GWAS p-value thresholds for SNPs.

|  | | AFB | | AFS | | AMC | | AMP | | NLB | |
| --- | --- | --- | --- | --- | --- | --- | --- | --- | --- | --- | --- |
| **PRS** | P-value threshold for SNPs | R^2^ | P | R^2^ | P | R^2^ | P | R^2^ | P | R^2^ | P |
| **ADHD** | 1 | 1.10E-02 | 1.20E-303 | 7.40E-03 | 3.30E-253 | 2.40E-05 | 4.20E-02 | 1.30E-03 | 3.20E-31 | 1.10E-03 | 9.70E-43 |
|  | 0.5 | 1.05E-02 | 1.06E-280 | 7.27E-03 | 1.06E-249 | 4.51E-05 | 5.25E-03 | 1.32E-03 | 3.18E-31 | 9.03E-04 | 8.21E-37 |
|  | 0.1 | 9.73E-03 | 1.85E-260 | 7.19E-03 | 3.70E-247 | 4.35E-05 | 6.09E-03 | 1.03E-03 | 1.09E-24 | 9.06E-04 | 6.36E-37 |
|  | 0.05 | 8.58E-03 | 1.09E-229 | 6.73E-03 | 3.38E-231 | 3.67E-05 | 1.18E-02 | 9.69E-04 | 2.20E-23 | 7.67E-04 | 1.68E-31 |
|  | 0.01 | 5.98E-03 | 1.86E-160 | 4.93E-03 | 6.78E-170 | 8.19E-06 | 2.34E-01 | 6.04E-04 | 3.60E-15 | 5.34E-04 | 1.90E-22 |
| **ASD** | 1 | 2.00E-04 | 7.60E-07 | 7.30E-05 | 7.10E-04 | 1.20E-04 | 5.30E-06 | 8.50E-05 | 3.30E-03 | 6.70E-05 | 5.70E-04 |
|  | 0.5 | 1.62E-04 | 9.15E-06 | 3.92E-05 | 1.33E-02 | 4.11E-05 | 7.71E-03 | 8.55E-05 | 3.08E-03 | 5.61E-05 | 1.59E-03 |
|  | 0.1 | 1.12E-04 | 2.24E-04 | 2.65E-05 | 4.18E-02 | 2.80E-05 | 2.78E-02 | 6.08E-05 | 1.26E-02 | 5.63E-05 | 1.56E-03 |
|  | 0.05 | 9.07E-05 | 9.02E-04 | 2.54E-05 | 4.66E-02 | 2.73E-05 | 2.97E-02 | 6.25E-05 | 1.14E-02 | 3.43E-05 | 1.35E-02 |
|  | 0.01 | 5.28E-05 | 1.13E-02 | 2.88E-05 | 3.38E-02 | 4.10E-05 | 7.77E-03 | 1.19E-04 | 4.78E-04 | 1.74E-05 | 7.88E-02 |
| **ED** | 1 | 4.60E-04 | 6.50E-14 | 1.90E-04 | 4.70E-08 | 9.00E-08 | 9.00E-01 | 3.90E-07 | 8.40E-01 | 6.10E-06 | 3.00E-01 |
|  | 0.5 | 3.19E-04 | 4.76E-10 | 2.06E-04 | 1.37E-08 | 4.95E-07 | 7.70E-01 | 1.13E-05 | 2.82E-01 | 1.89E-05 | 6.68E-02 |
|  | 0.1 | 2.66E-04 | 1.31E-08 | 1.84E-04 | 8.00E-08 | 9.10E-07 | 6.92E-01 | 1.51E-05 | 2.13E-01 | 2.87E-05 | 2.40E-02 |
|  | 0.05 | 2.78E-04 | 6.16E-09 | 1.60E-04 | 5.65E-07 | 6.84E-07 | 7.31E-01 | 2.09E-05 | 1.44E-01 | 4.30E-05 | 5.69E-03 |
|  | 0.01 | 1.26E-04 | 9.34E-05 | 9.05E-05 | 1.71E-04 | 9.48E-06 | 2.01E-01 | 1.30E-05 | 2.48E-01 | 5.50E-05 | 1.77E-03 |
| **BIP** | 1 | 3.20E-04 | 6.30E-10 | 5.50E-07 | 7.70E-01 | 2.40E-05 | 4.20E-02 | 3.10E-10 | 1.00E+00 | 6.70E-06 | 2.80E-01 |
|  | 0.5 | 3.45E-04 | 9.27E-11 | 7.37E-06 | 2.84E-01 | 3.27E-05 | 1.75E-02 | 5.17E-07 | 8.18E-01 | 1.22E-06 | 6.41E-01 |
|  | 0.1 | 3.44E-04 | 1.02E-10 | 1.02E-05 | 2.06E-01 | 2.77E-05 | 2.87E-02 | 2.43E-06 | 6.18E-01 | 5.70E-06 | 3.14E-01 |
|  | 0.05 | 2.71E-04 | 9.55E-09 | 6.14E-06 | 3.28E-01 | 4.09E-05 | 7.87E-03 | 7.99E-06 | 3.66E-01 | 7.79E-06 | 2.39E-01 |
|  | 0.01 | 1.33E-04 | 5.95E-05 | 2.86E-06 | 5.04E-01 | 1.73E-05 | 8.36E-02 | 4.44E-07 | 8.31E-01 | 8.45E-06 | 2.20E-01 |
| **MDD** | 1 | 5.90E-04 | 2.20E-17 | 3.60E-04 | 5.20E-14 | 5.60E-06 | 3.30E-01 | 1.80E-05 | 1.70E-01 | 8.20E-05 | 1.30E-04 |
|  | 0.5 | 5.41E-04 | 5.02E-16 | 2.98E-04 | 9.02E-12 | 1.01E-07 | 8.95E-01 | 3.37E-05 | 6.33E-02 | 4.02E-05 | 7.55E-03 |
|  | 0.1 | 3.99E-04 | 3.34E-12 | 1.95E-04 | 3.38E-08 | 7.30E-07 | 7.22E-01 | 2.28E-05 | 1.27E-01 | 3.61E-05 | 1.13E-02 |
|  | 0.05 | 3.27E-04 | 2.80E-10 | 1.94E-04 | 3.77E-08 | 1.49E-06 | 6.12E-01 | 2.88E-05 | 8.62E-02 | 4.29E-05 | 5.75E-03 |
|  | 0.01 | 1.70E-04 | 5.56E-06 | 1.24E-04 | 1.11E-05 | 1.98E-06 | 5.59E-01 | 4.72E-05 | 2.80E-02 | 2.55E-05 | 3.33E-02 |
| **SCZ** | 1 | 9.10E-05 | 8.90E-04 | 6.00E-04 | 3.50E-22 | 3.80E-06 | 4.20E-01 | 2.70E-05 | 9.80E-02 | 1.40E-04 | 7.20E-07 |
|  | 0.5 | 1.09E-04 | 2.75E-04 | 6.93E-04 | 2.40E-25 | 1.77E-05 | 8.05E-02 | 2.22E-05 | 1.32E-01 | 1.25E-04 | 2.33E-06 |
|  | 0.1 | 7.22E-05 | 3.05E-03 | 6.54E-04 | 5.00E-24 | 1.50E-05 | 1.08E-01 | 5.42E-05 | 1.85E-02 | 1.26E-04 | 2.31E-06 |
|  | 0.05 | 8.38E-05 | 1.42E-03 | 5.54E-04 | 1.41E-20 | 1.19E-05 | 1.51E-01 | 3.88E-05 | 4.63E-02 | 1.15E-04 | 5.96E-06 |
|  | 0.01 | 1.03E-04 | 4.04E-04 | 4.27E-04 | 3.22E-16 | 1.80E-05 | 7.73E-02 | 1.26E-05 | 2.57E-01 | 8.70E-05 | 8.40E-05 |

Dependent variables were adjusted for age at interview, year of birth, assessment centre at which the participant consented, genotype batch, and the first 15 principal components. The number of records used for the analyses was 121,544 for AFB, 156,143 for AFS, 102,386 for AMP, and 172,856 for AMC and 177,744 for NLB.

AFB: age at first firth. AFS: age first sexual intercourse. AMP: age at menopause. AMC: age at menarche. NLB: number of live births. ADHD: Attention-Deficit/Hyperactivity Disorder. ASD: Autism spectrum disorder. ED: Eating disorder. BIP: Bipolar disorder. MDD: Major depressive disorder. SCZ: Schizophrenia

## Table S11. Estimated genetic correlations between the five reproductive traits and the six psychiatric disorders.

|  |  | Genetic  correlation | SE | P-value | Genetic  correlation | SE | P-value |
| --- | --- | --- | --- | --- | --- | --- | --- |
|  |  | Base^a^ | | | Adv^b^ | | |
| **AFB** | ADHD | -0.677 | 0.034 | **1.86E-89** | -0.627 | 0.050 | **1.56E-35** |
|  | ASD | 0.160 | 0.056 | 4.40E-03 | 0.073 | 0.077 | 3.40E-01 |
|  | ED | 0.349 | 0.061 | **1.01E-08** | 0.410 | 0.080 | **2.64E-07** |
|  | BIP | 0.124 | 0.050 | 1.35E-02 | 0.001 | 0.069 | 9.84E-01 |
|  | MDD | -0.273 | 0.069 | **7.21E-05** | -0.260 | 0.091 | 4.16E-03 |
|  | SCZ | -0.040 | 0.029 | 1.75E-01 | -0.066 | 0.040 | 9.83E-02 |
| **AFS** | ADHD | -0.563 | 0.034 | **3.42E-60** | -0.481 | 0.039 | **2.18E-34** |
|  | ASD | 0.117 | 0.060 | 4.92E-02 | 0.055 | 0.074 | 4.57E-01 |
|  | ED | 0.189 | 0.055 | **6.53E-04** | 0.157 | 0.064 | 1.33E-02 |
|  | BIP | -0.047 | 0.048 | 3.22E-01 | -0.122 | 0.054 | 2.31E-02 |
|  | MDD | -0.265 | 0.066 | **6.49E-05** | -0.207 | 0.074 | 5.31E-03 |
|  | SCZ | -0.100 | 0.030 | **8.40E-04** | -0.114 | 0.035 | 1.01E-03 |
| **AMC** | ADHD | -0.272 | 0.038 | **5.71E-13** | -0.178 | 0.038 | **3.48E-06** |
|  | ASD | 0.126 | 0.060 | 3.51E-02 | 0.071 | 0.062 | 2.48E-01 |
|  | ED | 0.004 | 0.067 | 9.59E-01 | -0.024 | 0.071 | 7.35E-01 |
|  | BIP | -0.034 | 0.053 | 5.15E-01 | -0.028 | 0.056 | 6.13E-01 |
|  | MDD | -0.001 | 0.081 | 9.87E-01 | 0.077 | 0.081 | 3.44E-01 |
|  | SCZ | -0.028 | 0.030 | 3.42E-01 | -0.043 | 0.033 | 1.82E-01 |
| **AMP** | ADHD | 0.005 | 0.032 | 8.87E-01 | -0.020 | 0.032 | 5.25E-01 |
|  | ASD | -0.069 | 0.040 | 8.27E-02 | -0.067 | 0.041 | 1.08E-01 |
|  | ED | 0.017 | 0.048 | 7.20E-01 | 0.016 | 0.050 | 7.53E-01 |
|  | BIP | 0.040 | 0.040 | 3.21E-01 | 0.053 | 0.041 | 1.95E-01 |
|  | MDD | -0.079 | 0.058 | 1.76E-01 | -0.072 | 0.059 | 2.24E-01 |
|  | SCZ | 0.039 | 0.024 | 1.08E-01 | 0.042 | 0.025 | 9.22E-02 |
| **NLB** | ADHD | 0.356 | 0.042 | **4.01E-17** | 0.293 | 0.049 | **2.87E-09** |
|  | ASD | -0.072 | 0.066 | 2.71E-01 | -0.058 | 0.073 | 4.24E-01 |
|  | ED | -0.080 | 0.074 | 2.79E-01 | -0.047 | 0.081 | 5.61E-01 |
|  | BIP | 0.044 | 0.054 | 4.15E-01 | 0.104 | 0.060 | 8.07E-02 |
|  | MDD | 0.133 | 0.068 | 5.19E-02 | 0.142 | 0.080 | 7.59E-02 |
|  | SCZ | 0.075 | 0.031 | 1.54E-02 | 0.102 | 0.035 | 3.43E-03 |

^a^Dependent variables in Based model were adjusted for age at interview, year of birth, assessment centre at which the participant consented, genotype batch, the first 15 principal components, socioeconomic status, and smoking and alcohol consumption status.

^b^Dependent variables were further adjusted for educational and income level, and smoking and alcohol consumption status in Adv model.

AFB: age at first firth. AFS: age first sexual intercourse. AMP: age at menopause. AMC: age at menarche. NLB: number of live births. ADHD: Attention-Deficit/Hyperactivity Disorder. ASD: Autism spectrum disorder. ED: Eating disorder. BIP: Bipolar disorder. MDD: Major depressive disorder. SCZ: Schizophrenia
